# VAM-Based Equivalent Cauchy Model for Accordion Honeycomb Structures with Zero Poisson’s Ratio

**DOI:** 10.3390/ma18153502

**Published:** 2025-07-25

**Authors:** Yuxuan Lin, Mingfang Chen, Zhenxuan Cai, Zhitong Liu, Yifeng Zhong, Rong Liu

**Affiliations:** 1 School of Civil Engineering, Chongqing University, Chongqing 400045, China; linyx010212@163.com (Y.L.); chenminfang2025@163.com (M.C.); m13101329388@163.com (Z.C.); 20223449@stu.cqu.edu.cn (Z.L.); 20231601032@stu.cqu.edu.cn (R.L.); 2 Key Laboratory of New Technology for Construction of Cities in Mountain Area, Chongqing University, Chongqing 400045, China

**Keywords:** zero Poisson’s ratio, accordion honeycomb, energy absorption, tunable stiffness, variational asymptotic method, finite element analysis

## Abstract

The accordion honeycomb has unique deformation characteristics in cellular materials. This study develops a three-dimensional equivalent Cauchy continuum model (3D-ECM) based on the variational asymptotic method (VAM) to efficiently predict the mechanical response of the accordion honeycomb. The accuracy of the 3D-ECM is validated via quasi-static compression experiments on 3D-printed specimens and detailed 3D finite element simulations (3D-FEM), showing a strong correlation between simulation and experimental data. Parametric analyses reveal that the re-entrant angle, ligament-to-strut length ratio, and thickness ratios significantly affect the equivalent elastic moduli, providing insights into geometric optimization strategies for targeted mechanical performance. Comparative experiments among honeycomb structures with positive, negative, and zero Poisson’s ratios show that the accordion honeycomb achieves superior dimensional stability and tunable stiffness but exhibits lower energy-absorption efficiency due to discontinuous buckling and recovery processes. Further comparison among different ZPR honeycombs confirms that the accordion design offers the highest equivalent modulus in the re-entrant direction. The findings underscore the accordion honeycomb’s promise in scenarios demanding structural reliability, tunable stiffness, and moderate energy absorption.

## 1. Introduction

Lightweight cellular structures—particularly honeycombs—have emerged as a focal point of modern engineering design due to their exceptional energy-absorption and load-bearing capacities [1,2]. Such structures offer a desirable combination of low weight and high strength, and are widely used in aerospace, automotive, and protective systems where impact resistance and structural efficiency are critical [3,4]. Traditional honeycomb configurations (e.g., hexagonal, square, circular) have been extensively studied; however, their performance is often constrained by geometric limitations, leading to suboptimal energy absorption and non-uniform stress distribution under dynamic loads [5]. These limitations motivate the exploration of advanced cellular designs that can overcome the trade-offs faced by conventional honeycombs [6].

One major innovation is the development of auxetic and zero Poisson’s ratio honeycombs [7,8,9]. Auxetic honeycombs, which exhibit a negative Poisson’s ratio (NPR), contract laterally when compressed (“shrink-under-compression”), endowing them with superior energy-absorption capability ideal for impact protection and shock mitigation [10,11]. In contrast, zero Poisson’s ratio (ZPR) honeycombs maintain dimensional stability under compression with minimal lateral strain, meaning they experience virtually no transverse deformation when loaded [12,13]. This ZPR behavior is advantageous for applications requiring precision and deployability—for example, morphing aerospace structures or deployable mechanisms—where any lateral expansion could be detrimental [14].

**Figure 1:**
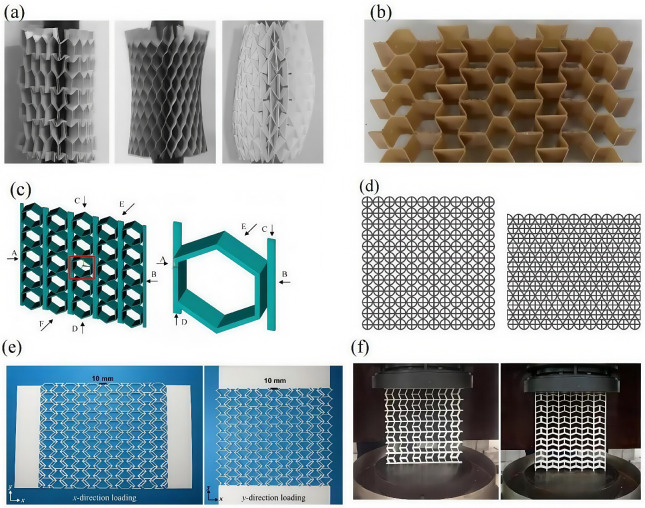
Novel ZPR honeycomb structures: (**a**) zero/positive/negative Poisson’s ratio hexagonal honeycomb compatibility with cylindrical structures [21], (**b**) positive and negative Poisson’s ratio compensation structure [24], (**c**) thin plate connection hexagonal ZPR structure with red box denoting the unit cell [28], (**d**) cross-circular ZPR structure [29], (**e**) fish-cell ZPR structure [30], and (**f**) parallelogram ZPR structure [31].

Recently, novel honeycomb topologies including re-entrant (auxetic) geometries [15], gradient-density cells [16], and hierarchical layouts [17,18] have been proposed to enhance mechanical performance beyond the traditional designs. Among these innovations, the ZPR honeycomb has emerged as a promising concept due to its unique deformation mechanisms and stable structural response under compression [19,20]. Grima et al. [21] proposed a semi-re-entrant hexagonal auxetic honeycomb in Figure 1a that demonstrates superior stiffness compared to conventional hexagonal and auxetic honeycombs, along with excellent compatibility with cylindrical structures. Huang et al. [22] developed a negative Poisson’s ratio structure based on parallel rod connections, while Chen et al. [23] designed a quasi-parallelogram auxetic configuration—both of which can be considered as approximate variants of square honeycombs. Broccolo et al. [24] introduced an innovative positive and negative Poisson’s ratio compensation structure in Figure 1b; however, this design fails to retain auxetic behavior under large axial tensile or compressive deformations. Later, Xu et al. [25,26] conducted comprehensive theoretical and experimental investigations on compensation honeycomb structures, revealing that these auxetic systems exhibit remarkably stable collapse modes due to their coordinated positive and negative Poisson’s ratio mechanisms.

Due to the superior properties of the zero Poisson’s ratio compensation mechanism, such structures have garnered increasing research interest. Recent advances, such as those incorporating shape memory and ring-like configurations enabled by 4D printing, have further expanded their functional potential [27]. Wu et al. [28] proposed the cross-circular zero Poisson’s ratio structure in Figure 1c, which exhibited exceptional energy-absorption performance. Huang et al. [29] developed a hexagonal auxetic structure interconnected by thin plates in Figure 1d, where two distinct components independently governed in-plane and out-of-plane deformations. An auxetic metamaterial inspired by fish cell configuration in Figure 1e,f was shown to achieve zero Poisson’s ratio along two orthogonal principal directions. This unique large-deformation mechanism holds promising potential for aerospace vehicle design [30,31]. However, conventional ZPR honeycombs still face a key challenge: it remains difficult to achieve simultaneously high energy absorption and high load-bearing efficiency in a single design [32]. In particular, many ZPR configurations show excellent stability but relatively limited energy-absorption capacity compared to auxetic counterparts [33]. This trade-off is especially pronounced under complex (e.g., multi-directional) loading conditions, making it clear that further innovation is needed to fully exploit ZPR structures in demanding applications [34].

To address these limitations, this study proposed the ZPR–accordion honeycomb structure, which integrates the zero Poisson’s ratio characteristic with an accordion-like folding mechanism [35,36]. The core idea is to combine the dimensional stability of ZPR honeycombs with a bio-inspired (or origami-inspired) accordion folding geometry to enable more efficient collapse mechanisms [37,38]. Such an accordion-inspired pattern facilitates progressive collapse and uniform stress distribution throughout the honeycomb, thereby mitigating the premature localized failure modes often observed in traditional honeycombs [39,40,41]. Furthermore, the accordion-type ZPR structure offers notable advantages in manufacturability, performance, and energy absorption. Its origami-inspired folding pattern enables efficient production through advanced methods like 3D printing, allowing for complex geometries with high mechanical performance. Compared to traditional ZPR structures, the accordion-type design provides superior dimensional stability, tunable stiffness, and enhanced energy absorption via a progressive collapse mechanism. These features make it ideal for applications requiring precise dimensional control and improved energy dissipation, such as in aerospace and protective systems.

Rather than emphasizing geometric innovation, the primary objective is to establish an efficient analytical framework that captures the mechanical behavior of such structures with reduced computational cost. First, a three-dimensional equivalent continuum model of the accordion honeycomb is developed using the variational asymptotic method (VAM), which systematically decomposes the original heterogeneous structure into a homogenized continuum representation. By introducing appropriate kinematic assumptions and asymptotic expansions of the displacement field, the model analytically derives the effective stiffness tensor and captures the anisotropic elastic response of the accordion honeycomb. This method offers significant advantages in reducing computational complexity, enabling rapid parametric studies without resorting to full-scale meshing of the intricate geometry.

This study focuses on the mechanical behavior of the honeycomb core under compressive loading. The analysis of the full sandwich composite structure, including both the core and face sheets, will be addressed in future work. The detailed 3D finite element simulations (3D-FEM) and quasi-static compression experiments are performed on additively manufactured (3D-printed) specimens to validate the model and evaluate the honeycomb’s performance under uniaxial loading. Furthermore, the influence of fundamental geometric parameters such as cell size and relative density is systematically investigated, as these dimensions critically affect the equivalent stiffness, deformation stability, and energy-absorption characteristics of the structure. Understanding their roles is essential for guiding structural optimization and ensuring scalable mechanical performance.

## 2. Geometric Design of ZPR–Accordion Honeycomb

**Figure 2:**
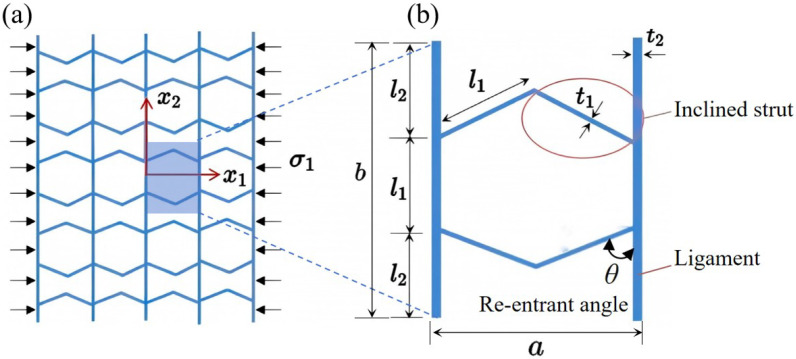
Schematic of (**a**) ZPR–accordion honeycomb and (**b**) its representative unit cell, highlighting key geometric parameters used in the design.

The ZPR–accordion honeycomb is designed by arranging inclined struts in an accordion-like configuration, enabling zero Poisson’s ratio deformation while enhancing the load-bearing capacity. The unit cell geometry is defined by six key parameters: length and thickness of the inclined strut ($l_{1}$ and $t_{1}$), length and thickness of the ligament ($l_{2}$ and $t_{2}$), vertical height of the unit cell (*h*), and re-entrant angle (θ). A schematic of the ZPR–accordion unit cell is shown in Figure 2, illustrating these geometric parameters.

To prevent overlap of structural elements in their undeformed configuration, the following geometric condition should hold:

(1) $$l_{1}\cos\theta <l_{2}$$

The equivalent density $\rho^{*}$ of the ZPR–accordion honeycomb is determined by dividing the unit cell’s mass by the total volume it occupies. The calculation is based on the detailed geometry of the struts and ligaments. The mass *m* of the ZPR–accordion unit cell is given by:

(2) $$m=\left[4l_{1}t_{1}+2l_{1}t_{2}+4l_{2}t_{2}\right]\times h\times \rho$$

where ρ denotes the base material’s density.

The unit cell occupies a total volume *V*, given by:

(3) V=b×a×h

where *b* and *a* are the projected dimensions defined as:

(4) $$\begin{array}{c} b=2l_{1}\sin\theta +t_{2}\\ a=2l_{2}+l_{1} \end{array}$$

Thus, the equivalent density $\rho^{*}$ can be expressed as:

(5) $$\rho^{*}=\frac{m}{V}=\frac{\left[4l_{1}t_{1}+2l_{1}t_{2}+4l_{2}t_{2}\right]\times \rho }{\left(2l_{2}+l_{1}\right)\left(2l_{1}\sin\theta +t_{2}\right)}$$

The derived density formulation was validated through experimental measurements under benchmark conditions, yielding a value of 0.163 g/cm^3^ compared to the measured 0.158 g/cm^3^, with a relative deviation of less than 5%. This expression captures the influence of key structural parameters—including strut lengths, ligament lengths, re-entrant angles, and strut thickness—on the lightweight efficiency of the ZPR–accordion honeycomb. It is particularly valuable for guiding structural design optimization under given density or weight constraints.

## 3. VAM-Based Equivalent Modeling of Accordion Honeycombs

**Figure 3:**
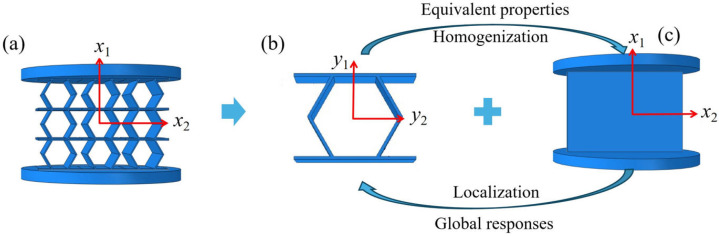
VAM-based equivalent modeling of accordion honeycombs, (**a**) 3D-FEM, (**b**) unit cell, and (**c**) 3D-ECM.

This section presents a three-dimensional equivalent Cauchy model (3D-ECM) for accordion honeycombs, developed through the Variational Asymptotic Method (VAM), as depicted in Figure 3. The deformation is analyzed under compressive load along the $x_{2}$-direction. The $x_{2}$, $x_{2}$, $x_{3}$ axes refer to the global coordinate system, while $y_{1}$, $y_{2}$, $y_{3}$ correspond to the local coordinate system. VAM-driven homogenization is employed to extract the effective macroscopic properties of the unit cell for macroscopic analysis. These global responses are then reintroduced into the cell model to enable localized field reconstruction via the established recovery relationships.

For the original accordion honeycomb, the partial derivative of displacement components $u_{i}$ can be obtained as

(6) $$\frac{\partial u_{i}\left(x_{i},y_{j}\right)}{\partial x_{i}}={\left.\frac{\partial u_{i}\left(x_{i},y_{j}\right)}{\partial x_{i}}\right|}_{y_{j}=\;\mathrm{const}\;}+{\left.\frac{1}{\zeta }\frac{\partial u_{i}\left(x_{i},y_{i}\right)}{\partial y_{j}}\right|}_{x_{i}=\;\mathrm{const}\;}\equiv u_{i,j}+\frac{1}{\zeta }u_{i;j},$$

where ζ is a small parameter representing the global-to-local ($y_{j}$ to $x_{i}$) scale ratio; i,j=1,2,3.

The virtual work principle yields the equilibrium equations governing the linear elastic behavior of the accordion honeycomb as

(7) $$\delta U=\bar{\delta W},$$

where δ denotes the Lagrange variation operator, *U* is the strain energy, $\bar{\delta W}$ corresponds to the virtual work of external forces, which need not necessarily derive from a potential functional.

Under the assumption of linear elasticity, the strain energy within the accordion honeycomb can be expressed as

(8) $$U=\int_{s}\frac{1}{\Omega }U_{\Omega }ds=\int_{s}\frac{1}{\Omega }\langle \frac{1}{2}\Gamma^{T}D\Gamma \rangle ds,$$

where $D\in M^{6}\left(R\right)$ is the material matrix; $\Omega \subset R^{3}\left(y_{i}\right)$ and $s\subset R^{3}\left(x_{i}\right)$ represent the respective coordinate domains at different scales.

**Figure 4:**
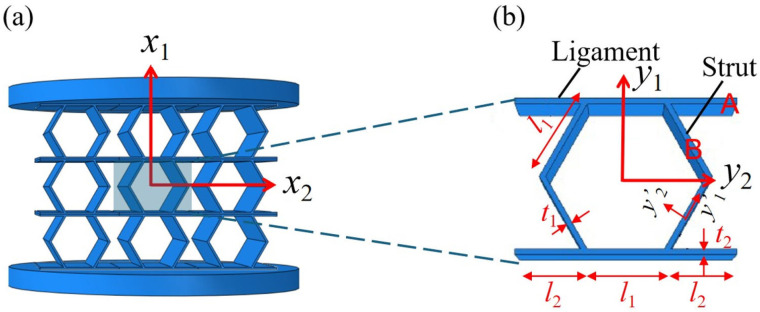
Partitioning of the accordion cell for strain energy integration: (**a**) 3 × 3 unit cells and (**b**) accordion cell.

For a periodic unit cell Ω with micro-coordinates $y_{j}$, the strain energy density is given by

(9) $$\begin{array}{cc} \; & U_{\Omega }=4\times \int_{\frac{-h_{2}}{2}}^{\frac{h_{h}}{2}}\int_{\frac{l_{n}^{2}}{2}}^{l_{2}+\frac{l_{1}}{2}}\int_{h_{1}\cos\theta }^{h_{1}\cos\theta +h_{2}}\Gamma_{A}^{T}D_{A}\Gamma_{A}dy_{1}dy_{2}dy_{3}\\ \; & +6\times \int_{\frac{-h_{2}}{2}}^{\frac{h_{h}}{2}}\int_{-\frac{t_{1}}{2}}^{\frac{\hbar_{t}}{2}}\int_{-\frac{h_{2}^{2}}{2}}^{\frac{h_{h}}{2}}\Gamma_{B}^{T}D_{B}\Gamma_{B}dy_{1}^{\prime }dy_{2}^{\prime }dy_{3}^{\prime } \end{array},$$

where subscripts *A* and *B* represent the corresponding segments in Figure 4; $y_{i}^{\prime }$ denotes the local element coordinates in the inclined strut, which are updated based on the movement and rotation of the cell’s struts. This in situ coordinate system enabled the tracking of deformation evolution at the local level, while preserving the global reference for the overall structural behavior under compression.

The three-dimensional strain field Γ of accordion honeycombs can be expressed in matrix notation as

(10) $$\Gamma =\Gamma_{h}w+\Gamma_{\epsilon }\bar{\epsilon }+\zeta \Gamma_{l}w,$$

where $\Gamma ={\left[\begin{array}{cccccc} \varepsilon_{11} & \varepsilon_{22} & \varepsilon_{33} & 2\varepsilon_{23} & 2\varepsilon_{13} & 2\varepsilon_{12} \end{array}\right]}^{T}$, $w={\left[\begin{array}{ccc} w_{1} & w_{2} & w_{3} \end{array}\right]}^{T}$ is the fluctuating function; $\bar{\epsilon }={\left[\begin{array}{cccccc} {\bar{\epsilon }}_{11} & {\bar{\epsilon }}_{22} & {\bar{\epsilon }}_{33} & 2{\bar{\epsilon }}_{23} & 2{\bar{\epsilon }}_{13} & 2{\bar{\epsilon }}_{12} \end{array}\right]}^{T}$, with ${\bar{\epsilon }}_{ij}$ denoting the Biot strain components, representing the macroscopic deformation in the Cauchy continuum framework; $\Gamma_{h}$, $\Gamma_{\epsilon }$, and $\Gamma_{l}$ are operator matrices,

(11) $$\Gamma_{h}=\left[\begin{array}{ccc} \frac{\partial }{\partial y_{1}} & 0 & 0\\ 0 & \frac{\partial }{\partial y_{2}} & 0\\ 0 & 0 & \frac{\partial }{\partial y_{3}}\\ 0 & \frac{\partial }{\partial y_{3}} & \frac{\partial }{\partial y_{2}}\\ \frac{\partial }{\partial y_{3}} & 0 & \frac{\partial }{\partial y_{1}}\\ \frac{\partial }{\partial y_{2}} & \frac{\partial }{\partial y_{1}} & 0 \end{array}\right],\Gamma_{\epsilon }=\left[\begin{array}{cccccc} 1 & 0 & 0 & 0 & 0 & 0\\ 0 & 1 & 0 & 0 & 0 & 0\\ 0 & 0 & 1 & 0 & 0 & 0\\ 0 & 0 & 0 & 1 & 0 & 0\\ 0 & 0 & 0 & 0 & 1 & 0\\ 0 & 0 & 0 & 0 & 0 & 1 \end{array}\right],\Gamma_{l}=\left[\begin{array}{ccc} \frac{\partial }{\partial x_{1}} & 0 & 0\\ 0 & \frac{\partial }{\partial x_{2}} & 0\\ 0 & 0 & \frac{\partial }{\partial x_{3}}\\ 0 & \frac{\partial }{\partial x_{3}} & \frac{\partial }{\partial x_{2}}\\ \frac{\partial }{\partial x_{3}} & 0 & \frac{\partial }{\partial x_{1}}\\ \frac{\partial }{\partial x_{2}} & \frac{\partial }{\partial x_{1}} & 0 \end{array}\right]$$

The external work contribution $\bar{\delta W}$ in the principle of virtual work is given by

(12) $$\bar{\delta W}={\bar{\delta W}}_{H}+\zeta {\bar{\delta W}}^{*},$$

where ${\bar{\delta W}}_{H}$ represents the macroscopic work contribution at the homogenized scale, and ${\bar{\delta W}}^{*}$ accounting for the microstructural fluctuation effects,

(13) $${\bar{\delta W}}_{H}=\int \left(f_{i}{\bar{\delta q}}_{i}+m_{i}{\bar{\delta \psi }}_{i}\right)ds,\;{\bar{\delta W}}^{*}=\int \frac{1}{\Omega }\left(\langle p_{i}\delta w_{i}\rangle +\oint Q_{i}\delta w_{i}d\partial \Omega \right)ds,$$

where ${\bar{\delta q}}_{i}$ and $\bar{\delta \psi_{i}}$ represent virtual linear and angular displacements, respectively; $p_{i}$ and $Q_{i}$ are the surface tractions and distributed body forces;

(14) $$f_{i}=\frac{1}{\Omega }\left(\langle p_{i}\rangle +\oint Q_{i}d\partial \Omega \right),m_{i}=\frac{e_{i\alpha j}}{\Omega }\left(\langle \zeta y_{\alpha }p_{j}\rangle +\oint \zeta y_{\alpha }Q_{j}d\partial \Omega \right),$$

with $e_{i\alpha j}$ denoting the third-order Levi-Civita permutation tensor in $R^{3}$.

If $Q_{i}$ and $p_{i}$ are independent of the microscopic fluctuations, the fluctuation-associated virtual work ${\bar{\delta W}}^{*}$ reduces to

(15) $${\bar{\delta W}}^{*}=\delta \int \frac{1}{\Omega }\left(\langle p_{i}w_{i}\rangle +\oint Q_{i}w_{i}d\partial \Omega \right)ds.$$

Integrating the strain energy formulation in Equation (8) with the virtual work terms from Equations (12) and (13), and substituting them into the variational expression of Equation (7), yields the governing equilibrium equations,

(16) $$\int \frac{1}{\Omega }\delta \left[\frac{1}{2}\langle \Gamma^{T}D\Gamma \rangle -\zeta \left(\langle p_{i}w_{i}\rangle +\oint Q_{i}w_{i}d\partial \Omega \right)\right]-\left(f_{i}{\bar{\delta q}}_{i}+m_{i}{\bar{\delta \psi }}_{i}\right)ds=0.$$

The coupling between microscopic and macroscopic coordinates through the fluctuation field $w_{i}$ introduces significant modeling complexity. While conventional homogenization approaches often neglect these fluctuations to obtain a simplified continuum representation, such simplification may yield substantial errors for microstructures exhibiting strong anisotropic behavior. The VAM enables systematic derivation of the fluctuation field solution via asymptotic analysis of the variational principle in Equation (16).

Given the independence of the last two terms in Equation (16) from the fluctuation field $w_{i}$, the governing variational equation for the microscopic displacement field can be isolated as

(17) $$\delta \left[\frac{1}{2}\langle \Gamma^{T}D\Gamma \rangle -\zeta \left(\langle p_{i}w_{i}\rangle +\oint Q_{i}w_{i}d\partial \Omega \right)\right]=0.$$

Through consistent asymptotic expansion while discarding O(ζ) and smaller terms in the VAM framework, the simplified variational expression of Equation (17) becomes

(18) $$\delta \frac{1}{2}\langle {\left(\Gamma_{h}w+\Gamma_{\epsilon }\bar{\epsilon }\right)}^{T}D\left(\Gamma_{h}w+\Gamma_{\epsilon }\bar{\epsilon }\right)\rangle =0.$$

Numerical solution of Equation (18) typically requires discretization methods like the finite element approach, where the fluctuation field w is approximated through nodal values N and element-specific shape function *S*, such as

(19) $$w\left(x_{i},y_{j}\right)=S\left(y_{j}\right)N\left(x_{i}\right).$$

By incorporating the finite element discretization from Equation (19) into the variational principle Equation (18), the discrete strain energy expression becomes

(20) $$U=\frac{1}{2}\left(N^{T}D_{hh}N+2N^{T}D_{h\epsilon }\bar{\epsilon }+{\bar{\epsilon }}^{T}D_{\epsilon \epsilon }\bar{\epsilon }\right),$$

where

(21) $$D_{hh}=\langle {\left(\Gamma_{h}S\right)}^{T}D\left(\Gamma_{h}S\right)\rangle ,\;D_{h\epsilon }=\langle {\left(\Gamma_{h}S\right)}^{T}D\Gamma_{\epsilon }\rangle ,\;D_{\epsilon \epsilon }=\langle \Gamma_{\epsilon }^{T}D\Gamma_{\epsilon }\rangle .$$

Minimization of the discrete strain energy *U* in Equation (20) with respect to nodal parameters generates the constrained linear system, such as

(22) $$D_{hh}N=-D_{h\epsilon }\bar{\epsilon }.$$

Evidently, the nodal solution vector N scales linearly with the applied strain $\bar{\epsilon }$, admitting the symbolic representation as

(23) $$N=N_{0}\bar{\epsilon }.$$

The strain energy evaluation is achieved by substituting the symbolic solution of Equation (23) into the functional form given by Equation (20), resulting in

(24) $$U=\frac{1}{2}{\bar{\epsilon }}^{T}\left(N_{0}^{T}D_{h\epsilon }+D_{\epsilon \epsilon }\right)\bar{\epsilon }\equiv \frac{\Omega }{2}{\bar{\epsilon }}^{T}D_{e}^{*}\bar{\epsilon }.$$

where $D_{e}^{*}\in R^{6\times 6}$ denotes the effective stiffness matrix characterizing the material behavior under the Cauchy continuum framework.

The corresponding constitutive equation for the 3D-ECM, valid within the regime of linear elasticity for accordion honeycombs, takes the form:

(25) $$\left\{\begin{array}{c} \sigma_{11}\\ \sigma_{22}\\ \sigma_{33}\\ \sigma_{23}\\ \sigma_{13}\\ \sigma_{12} \end{array}\right\}=\left[\begin{array}{cccccc} C_{11} & C_{12} & C_{13} & C_{14} & C_{15} & C_{16}\\ C_{12} & C_{22} & C_{23} & C_{24} & C_{25} & C_{26}\\ C_{13} & C_{23} & C_{33} & C_{34} & C_{35} & C_{36}\\ C_{14} & C_{24} & C_{34} & C_{44} & C_{45} & C_{46}\\ C_{15} & C_{25} & C_{35} & C_{45} & C_{55} & C_{56}\\ C_{16} & C_{26} & C_{36} & C_{46} & C_{56} & C_{66} \end{array}\right]\left\{\begin{array}{c} \varepsilon_{11}\\ \varepsilon_{22}\\ \varepsilon_{33}\\ 2\varepsilon_{23}\\ 2\varepsilon_{13}\\ 2\varepsilon_{12} \end{array}\right\}.$$

where [C] represents the 6×6 stiffness matrix of the Cauchy continuum model, and $\varepsilon_{ij},\left(i,j=1,2,3\right)$ indicate the corresponding Cauchy strain components.

In the case of orthotropic materials, Equation (25) may be restructured as

(26) $$\left\{\begin{array}{c} \varepsilon_{11}\\ \varepsilon_{22}\\ \varepsilon_{33}\\ 2\varepsilon_{23}\\ 2\varepsilon_{13}\\ 2\varepsilon_{12} \end{array}\right\}=\left[\begin{array}{cccccc} \frac{1}{E_{1}} & -\frac{\nu_{21}}{E_{2}} & -\frac{\nu_{31}}{E_{3}} & 0 & 0 & 0\\ -\frac{\nu_{12}}{E_{1}} & \frac{1}{E_{2}} & -\frac{\nu_{32}}{E_{3}} & 0 & 0 & 0\\ -\frac{\nu_{13}}{E_{1}} & -\frac{\nu_{23}}{E_{2}} & \frac{1}{E_{3}} & 0 & 0 & 0\\ 0 & 0 & 0 & \frac{1}{G_{23}} & 0 & 0\\ 0 & 0 & 0 & 0 & \frac{1}{G_{13}} & 0\\ 0 & 0 & 0 & 0 & 0 & \frac{1}{G_{12}} \end{array}\right]\left\{\begin{array}{c} \sigma_{11}\\ \sigma_{22}\\ \sigma_{33}\\ \sigma_{23}\\ \sigma_{13}\\ \sigma_{12} \end{array}\right\}.$$

where $E_{1},E_{2}$, and $E_{3}$ are equivalent elastic moduli in three direction, $G_{12},G_{13}$, and $G_{23}$ correspond to the shear moduli in the 1–2, 1–3, and 2–3 planes, respectively, $\nu_{12},\nu_{13},\nu_{23}$ are equivalent Poisson’s ratios, and their reciprocal counterparts $\nu_{21},\nu_{31},\nu_{32}$ can be derived from the former using symmetry relations as

(27) $$\nu_{21}=\nu_{12}\frac{E_{2}}{E_{1}},\;\nu_{31}=\nu_{13}\frac{E_{3}}{E_{1}},\;\nu_{32}=\nu_{23}\frac{E_{3}}{E_{2}}$$

By inverting Equation (26), one retrieves the equivalent form presented in Equation (25) as

(28) $$\begin{array}{ccc} C_{11}=\frac{E_{1}\left(1-\nu_{23}\nu_{32}\right)}{\Delta }, & C_{12}=\frac{E_{2}\left(\nu_{12}+\nu_{13}\nu_{32}\right)}{\Delta }, & C_{13}=\frac{E_{3}\left(\nu_{13}+\nu_{12}\nu_{23}\right)}{\Delta },\\ C_{22}=\frac{E_{2}\left(1-\nu_{13}\nu_{31}\right)}{\Delta }, & C_{23}=\frac{E_{3}\left(\nu_{23}+\nu_{13}\nu_{21}\right)}{\Delta }, & C_{33}=\frac{E_{3}\left(1-\nu_{12}\nu_{21}\right)}{\Delta },\\ C_{44}=G_{23}, & C_{55}=G_{13}, & C_{66}=G_{12}, \end{array}$$

with $\Delta =1-\nu_{12}\nu_{21}-\nu_{23}\nu_{32}-\nu_{13}\nu_{31}-2\nu_{21}\nu_{13}\nu_{32}$ and other items being zero.

The current 3D-ECM focuses on linear elastic behavior under small strains, as validated in Section 4 and Section 5. While the VAM framework can be extended to geometric nonlinearity (e.g., large rotations), material nonlinearity and finite strain effects require additional kinematic assumptions and will be addressed in future work.

## 4. Experimental Design and Finite Element Modeling

**Figure 5:**
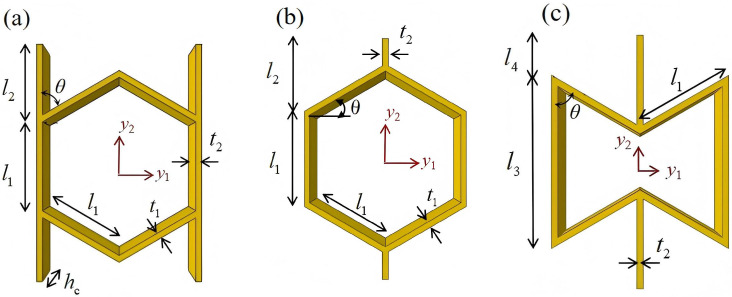
Schematic diagram of unit cells with different Poisson’s ratio: (**a**) accordion cell with near-zero Poisson’s ratio, (**b**) hexagonal cell with positive Poisson’s ratio, and (**c**) re-entrant cell with negative Poisson’s ratio.

This section investigates the static mechanical properties of three representative honeycomb structures: hexagonal honeycomb with positive Poisson’s ratio, re-entrant honeycomb with negative Poisson’s ratio, and accordion honeycomb with near-zero Poisson’s ratio, as illustrated in Figure 5. To ensure the comparability of results and eliminate the influence of structural size effects, all honeycomb structures have identical length, width, and height dimensions (see Table 1 for detailed geometric parameters).

### 4.1. Mechanical Property Testing of Specimen Materials

To accurately characterize the mechanical properties of the base material used for 3D-printed honeycomb specimens, standard tensile tests were conducted on acrylonitrile butadiene styrene (ABS) specimens fabricated by fused deposition modeling (FDM) technology. Dumbbell-shaped samples were printed using a Ruiku T5060 3D printer, manufactured by Ruiku Technology in Shenzhen, China, with 1.75 mm diameter ABS filaments. The linear accuracy of the printer is ±0.1 mm, and the repeatability is ±0.05 mm. All specimens were produced according to international standards, with a uniform thickness of 5 mm and a raster angle of 45°, adopting a vertical build orientation to ensure consistency in material properties. Following fabrication, all specimens underwent careful visual inspection and optical microscopy (20× magnification) examination. No significant porosity, warping, or structural anomalies were detected.

**Figure 6:**
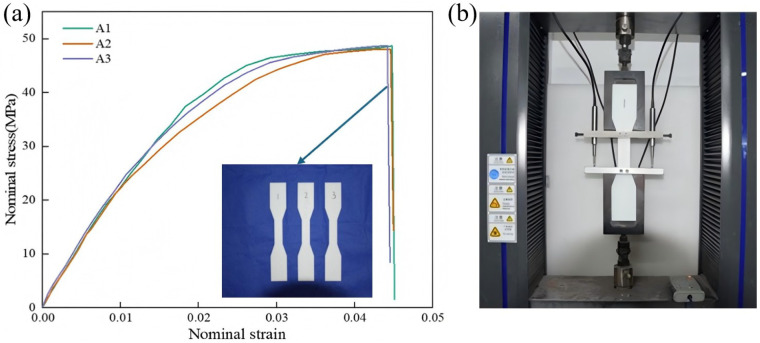
Tensile test results and testing setup, (**a**) nominal stress–strain curves of ABS material, (**b**) testing machine setup.

Tensile testing was performed using a universal testing machine (20 kN capacity, ±1% force accuracy) at a constant crosshead rate of 1 mm/min until failure, in accordance with ASTM D638 [42] (Figure 6). To ensure statistical reliability, three replicate tests were performed under identical conditions. The resulting nominal stress–strain curves exhibited consistent linear elastic behavior followed by plastic deformation prior to failure. The tensile test data were analyzed using OriginPro (Version 2022) for stress–strain curve fitting, and mechanical properties, including Young’s modulus, and fracture strain, were derived from the experimental results. The calculated values were: Young’s modulus of 2300 MPa, Poisson’s ratio of 0.389, density of 1.319 g/cm^3^, and ultimate strength of 45 MPa. The standard deviations for the elastic modulus and tensile strength measured from three tensile specimens were 85 MPa and 3.2 MPa, respectively. These properties were then used as material inputs for all finite element analyses in this study.

### 4.2. Quasi-Static Compressive Testing

**Figure 7:**
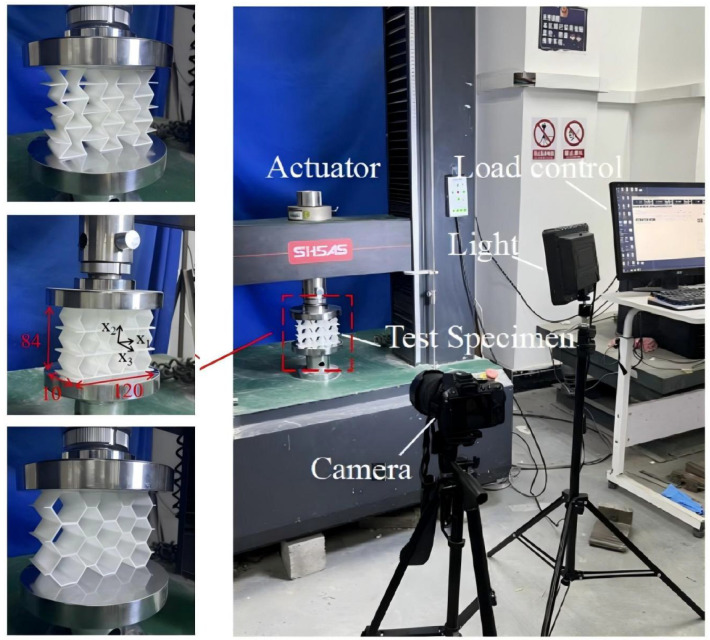
Uniaxial compression test configuration, including honeycomb specimen dimensions (in mm) and experimental setup, which features the testing machine, load cell, and imaging camera.

The 3 × 3 cellular honeycomb specimens were fabricated using fused deposition modeling (FDM) 3D printing technology with a layer resolution of 0.2 mm. The honeycomb structures were designed using SolidWorks 2019, and the models were sliced using Cura 4.8. Compression testing was performed on a WDW100 universal testing machine (Sunlight Group) equipped with a 100 kN load cell (±0.01 N resolution) and rigid parallel platens. The entire deformation process was recorded using high-definition digital imaging, with the experimental setup illustrated in Figure 7. The specimen was centered on the fixed bottom platen to ensure stability during testing. Quasi-static compression was applied at a constant rate of 1 mm/min until reaching a maximum displacement of 34 mm (corresponding to $\varepsilon_{22}=0.40$). Owing to the structural symmetry and force equilibrium of the honeycomb specimens, boundary effects—such as bending, torsion, or shear—were negligible during loading.

### 4.3. Finite Element Modeling

**Figure 8:**
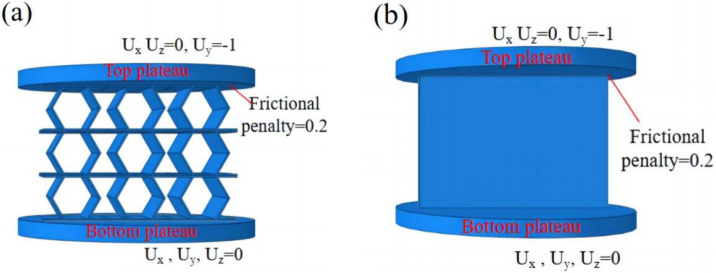
Boundary and loading conditions for (**a**) 3D-FEM and (**b**) 3D-ECM.

To validate the effectiveness of the 3D-ECM developed for the honeycomb structures, finite element simulations were conducted and compared against experimental results. Two numerical models were established using ABAQUS/Explicit solver (Version 2023): (1) a detailed 3D-FEM featuring explicit cellular geometry; (2) a homogenized 3D-ECM utilizing the derived equivalent stiffness matrix. The geometric parameters of the ZPR–accordion honeycomb were adopted from Table 1. The 3D-FEM consisted of 23,426 C3D4 tetrahedral elements, while the 3D-ECM employed 6674 C3D8R hexahedral elements. Material properties for the 3D-FEM were assigned based on tensile test results in Section 4.1, while the 3D-ECM incorporated the equivalent engineering constants derived from unit-cell homogenization in Equation (26), as summarized in Table 2. Both models applied identical boundary conditions to replicate the experimental configuration, illustrated in Figure 8a,b.

The 3D-ECM developed in this study is currently validated within the elastic regime. It is intended primarily for use in stiffness-driven design and optimization scenarios. Future extensions will focus on incorporating plastic and damage behaviors to broaden its applicability to failure prediction and structural reliability analysis.

## 5. Results and Discussion

### 5.1. Deformation Modes

Table 3 compares the compressive deformation processes of three honeycomb structures, highlighting the distinct collapse behaviors governed by their respective Poisson’s ratios. The accordion honeycomb, with zero Poisson’s ratio, maintains dimensional stability with negligible lateral displacement and is dominated by uniform axial bending of the unit cells, preserving structural integrity throughout compression. The re-entrant honeycomb, exhibiting negative Poisson’s ratio, contracts laterally by 18% at $\varepsilon_{22}$ = 0.4 and forms sequential “X”-shaped collapse bands, developing continuous plastic hinges with inter-cell contact. Meanwhile, the hexagonal honeycomb, with a positive Poisson’s ratio, undergoes lateral expansion by about 22% at $\varepsilon_{22}$ = 0.4, promoting a broader distribution of loads and mitigates localized failure.

**Figure 9:**
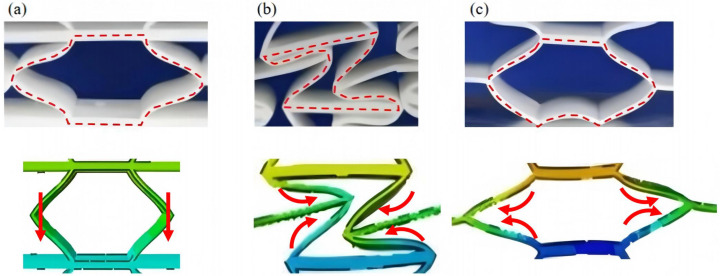
Typical deformation modes of honeycomb cells with different Poisson’s ratios: (**a**) ZPR–accordion cell, (**b**) NPR-re-entrant cell, and (**c**) PPR-hexagon cell.

Figure 9 demonstrates the characteristic deformation mechanisms of honeycomb cells with different Poisson’s ratio, as obtained from experiment and finite element simulation. The ZPR–accordion cell deforms through an accordion-like folding mechanism of its inclined struts, exhibiting exceptional transverse dimensional stability with minimal variation. In contrast, the NPR-re-entrant cell undergoes deformation via inward rotation and bending of inclined struts, resulting in significant lateral contraction while developing characteristic plastic hinges. Conversely, the PPR-hexagon cell displays outward rotation and bending of inclined struts, producing transverse expansion accompanied by prominent buckling deformation in the horizontal struts.

These distinct deformation modes yield significant mechanical consequences: the hexagonal honeycomb demonstrates superior load-bearing capacity (approximately 40% greater than the accordion honeycomb), while the re-entrant honeycomb maximizes energy absorption through its progressive collapse mechanism. The accordion honeycomb maintains exceptional dimensional stability under compressive loading. Collectively, these results highlight the pivotal influence of Poisson’s ratio on three key aspects: (1) collapse initiation, (2) deformation-localization patterns, and (3) load-redistribution characteristics. These findings provide crucial guidelines for selecting optimal cellular geometries tailored to specific engineering applications.

### 5.2. Nominal Stress–Strain Curves and Energy-Absorption Metrics

**Figure 10:**
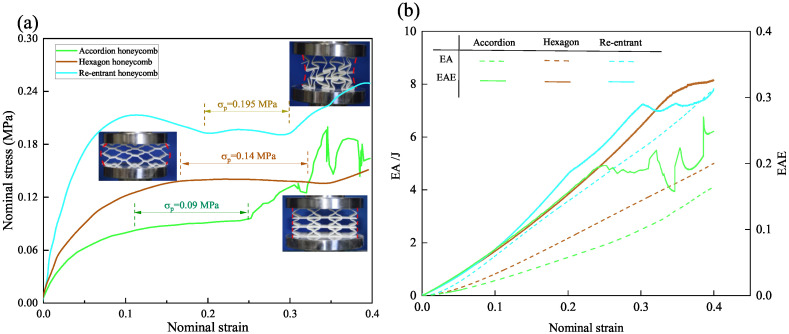
Comparison of (**a**) nominal stress–strain curves and (**b**) EA/EAE-strain curves for honeycomb structures with different Poisson’s ratio. Data shown represent a single experimental trial.

Figure 10a compares the compressive stress–strain curves of three honeycomb structures with different Poisson’s ratios under quasi-static compression. All three honeycomb structures demonstrate characteristic elastic-plateau-densification deformation behavior. During the elastic deformation stage, the re-entrant honeycomb exhibits the highest initial stiffness, with a deformation modulus of 3.9 MPa—approximately 20% and 40% greater than those of the hexagonal honeycomb (3.2 MPa) and accordion honeycomb (2.8 MPa), respectively. This enhanced stiffness originates from the re-entrant structure’s distinctive deformation mechanism: cell rotation coupled with lateral contraction facilitates the development of distributed plastic hinges, enabling effective load transfer while minimizing stress concentrations. In comparison, the hexagonal honeycomb’s stiffness derives mainly from rigid strut support, leading to more localized deformation patterns. The accordion honeycomb, constrained by its near-zero lateral deformation capability, demonstrates the lowest initial stiffness of the three structures.

In the plateau stage, the distinctive mechanical responses of each honeycomb structure become increasingly evident. To quantitatively characterize the energy-absorption capability, the plateau stress $\sigma_{p}$ is calculated as:

(29) $$\sigma_{p}=\frac{\int_{\varepsilon_{0}}^{\varepsilon_{d}}\sigma \left(\varepsilon \right)d\varepsilon }{\varepsilon_{d}-\varepsilon_{0}},$$

where $\varepsilon_{0}$ represents the strain at which the plateau stage begins, $\varepsilon_{d}$ is the densification strain, and σ(ε) is the instantaneous stress at strain ε. A summary of $\varepsilon_{0}$ and $\varepsilon_{d}$ values is listed in Table 4.

The re-entrant honeycomb achieves the highest plateau stress of 0.195 MPa, followed by the hexagonal honeycomb at 0.14 MPa, and the accordion honeycomb at 0.09 MPa. The hexagonal honeycomb benefits from synergistic lateral expansion, which enhances structural stability and promotes a more uniform stress distribution, thereby extending the plateau region. By contrast, the accordion honeycomb, lacking lateral deformation, undergoes concentrated longitudinal buckling, leading to a shorter plateau duration. During the plateau region, the accordion structure exhibits non-monotonic stress–strain behavior. This behavior can be attributed to localized buckling and stiffness recovery in certain regions of the structure. As the compression progresses, some struts undergo partial yielding, while other struts recover their stiffness, resulting in a non-monotonic characteristic observed in the stress–strain curve.

During the densification stage, all three structures exhibit a sharp increase in load-bearing capacity. However, the accordion honeycomb displays localized plastic deformation, resulting in transient stiffness degradation followed by recovery as densification progresses. This behavior clearly reveals a unique pattern of discontinuous stiffness restoration in the accordion honeycomb.

The energy-absorption (EA) curves in Figure 10b provide further insight into the role of Poisson’s ratio in governing energy-absorption performance. Energy absorption (EA), a key metric for assessing structural performance under compressive loading, quantifies the total energy absorption during deformation. The energy-absorption efficiency (EAE), on the other hand, assesses the structure’s ability to absorb energy relative to its deformation. These parameters are expressed as:

(30) $$EA=V\int_{0}^{\varepsilon_{d}}\sigma_{r}d\varepsilon ,\;\eta =\frac{\int_{0}^{\varepsilon_{r}}\sigma \left(\varepsilon \right)d\varepsilon }{{\left.\sigma (\varepsilon )\right|}_{\varepsilon =\varepsilon_{r}}},$$

where *V* is the volume occupied by the honeycomb structure, $\varepsilon =\varepsilon_{r}$ is the real-time nominal strain value.

In the early elastic stage, all three structures exhibit similar EAE values, as their response is primarily governed by elastic deformation. However, with increasing strain, the re-entrant honeycomb achieves the highest EAE due to its controlled plastic hinge formation and more uniform cell collapse mechanism. The hexagonal honeycomb, leveraging its extended plateau stage, exhibits a consistently rising EAE with minimal fluctuations. In contrast, the accordion honeycomb displays the lowest EAE, accompanied by pronounced fluctuations resulting from unstable buckling and intermittent stiffness recovery during compression.

These findings highlight the distinct energy-absorption characteristics of each structure: the re-entrant honeycomb excels in energy absorption and load distribution, the hexagonal honeycomb provides stable and prolonged energy absorption, while the accordion honeycomb prioritizes dimensional stability at the cost of reduced energy-absorption efficiency.

### 5.3. Finite Element Simulation Verification

**Figure 11:**
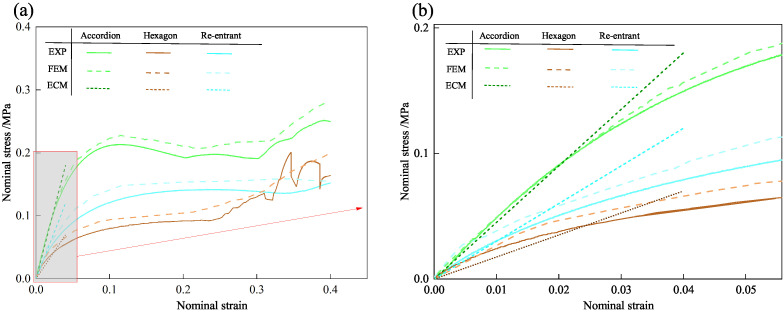
Comparison of (**a**) nominal stress–strain curves, with the shaded area denoting the enlarged region, and (**b**) enlarged elastic stress–strain curves obtained from quasi-static compression test and numerical simulation.

Figure 11 compares the nominal stress–strain curves obtained from three different models. Since this study primarily focuses on evaluating the accuracy of the equivalent model within the elastic stage, the stress–strain curve of the 3D-ECM is presented only up to the elastic stage. The 3D-ECM curves of three honeycomb structures exhibit the steepest slope, indicating the highest predicted stiffness. In contrast, the experimental data show the smallest slope, reflecting the relatively lower load-bearing capacity of the physical specimens. To quantify the accuracy of FEM and ECM models, Table 5 summarizes the elastic modulus obtained by experimental testing, finite element simulation, and equivalent continuum modeling. The FEM results deviated from experimental data by less than 4%, while ECM results showed less than 10% deviation, meeting typical engineering accuracy requirements. To quantitatively assess simulation accuracy, we calculated RMSE between FEM/ECM and experimental stress–strain data over the 0–5% strain interval. FEM predictions yield lower errors (<0.008 MPa RMSE), while ECM errors remain acceptably small (<0.014 MPa), supporting the <10% deviation claim.

The observed discrepancies can be attributed to the following factors: (1) manufacturing imperfections in 3D printing: the actual mechanical properties of the printed specimens may be slightly inferior to theoretical expectations due to factors such as weak interlayer adhesion and material deposition inconsistencies. (2) idealized assumptions in finite element simulations: in numerical models, loading and boundary conditions are assumed to be ideal, whereas minor misalignments, contact friction, and other practical factors during physical testing can influence the measured values. (3) simplifications in the homogenized model: the 3D-ECM is developed based on the variational asymptotic homogenization method, which inevitably neglects certain local details. Nevertheless, it captures the overall mechanical behavior of the structure with high fidelity.

To further validate the accuracy of the finite element models, Table 6 presents displacement contours predicted by 3D-FEM and 3D-ECM for three honeycomb configurations exhibiting varying Poisson’s ratios under 25 mm compressive loading. The predicted deformation patterns from both models align well with experimental data, confirming the robustness of the developed equivalent model. The close agreement between the 3D-ECM predictions and the experimental results confirms that the model can effectively support the engineering design and rapid analysis of honeycomb structures. However, it should be noted that the 3D-ECM homogenizes the honeycomb as a continuous medium and can only capture the overall macroscopic displacement trends. It is unable to reflect the detailed response characteristics of individual structural elements.

Deformation in the accordion honeycomb predominantly localizes within the inclined struts, aligning well with experimental findings. Moreover, the transverse displacements of the accordion honeycomb remain nearly zero, confirming the zero Poisson’s ratio characteristic—that is, the structure exhibits no lateral expansion or contraction under axial compression. The 3D-ECM not only maintains high predictive accuracy but also significantly reduces computational cost, making it particularly suitable for engineering optimization and large-scale structural analyses.

### 5.4. Evaluation of Computational Effectiveness

To further assess the practicality of the proposed equivalent modeling approaches, the computational efficiencies of the 3D-FEM and 3D-ECM under uniaxial compression were evaluated. The 3D-FEM employed ten-node tetrahedral elements (C3D10), while the 3D-ECM was discretized with eight-node reduced-integral hexahedral elements (C3D8R). To verify mesh convergence, we conducted a sensitivity analysis based on mesh size. Differences in elastic modulus and peak stress between 0.50 mm and 0.25 mm mesh sizes were below 2%, validating the mesh independence of ECM results. A mesh size of 0.50 mm was used throughout the study to balance accuracy and computational cost. Table 7 summarizes the element types, total element numbers, node numbers, and computational times.

Owing to the intricate cellular structure, 3D-FEM necessitated much finer meshing to capture local deformations accurately, leading to a markedly increased number of elements and nodes relative to the equivalent models. For the uniaxial compression simulation, the 3D-FEM involved 235,285 elements and 455,742 nodes, whereas the 3D-ECM model reduced these numbers to 77,792 elements and 83,835 nodes, respectively. Moreover, the 3D-ECM completed the analysis in 82 s, requiring only 14.4% of the computation time needed by the 3D-FEM (570 s).

These results demonstrate that the VAM-based equivalent model significantly reduces computational cost while maintaining sufficient accuracy for engineering applications. The 3D-ECM is particularly suitable for large-scale simulations where full geometric modeling would otherwise be computationally prohibitive. Overall, the VAM-based homogenization strategy provides a practical balance between predictive fidelity and computational efficiency, making it highly advantageous for the preliminary design and optimization of cellular metamaterials such as the ZPR–accordion honeycomb.

## 6. Parameter Analysis

The equivalent elastic properties of the ZPR accordion honeycomb structure were systematically investigated by varying four key geometric parameters (Table 8): the ligament-to-strut length ratio ($l_{2}/l_{1}$), the strut slenderness ratio ($l_{1}/t_{1}$), the ligament slenderness ratio ($l_{2}/t_{2}$), and the re-entrant angle (θ). Homogenization-based finite element analyses were conducted to evaluate their effects on the equivalent elastic moduli ($E_{1}$, $E_{2}$) and shear modulus ($G_{12}$). The cell geometries with different parameters are illustrated in Table 9.

### 6.1. Effect of Re-Entrant Angle

**Figure 12:**
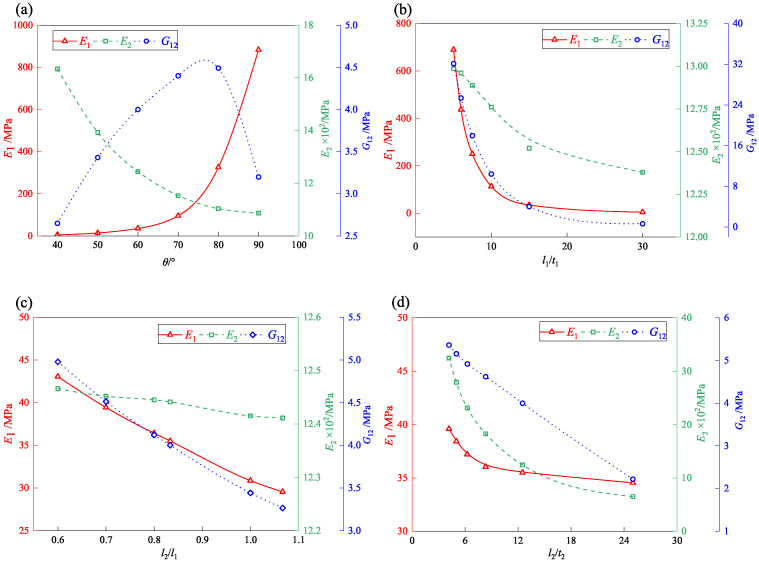
Effect of (**a**) θ, (**b**) $l_{1}/t_{1}$, (**c**) $l_{2}/l_{1}$, and (**d**) $l_{2}/t_{2}$ on equivalent elastic moduli and equivalent shear modulus in the accordion honeycomb.

Figure 12a illustrates that varying the re-entrant angle (θ) from 40° to 90° significantly influences the equivalent elastic modulus $E_{1}$. For θ between 40° and 60°, $E_{1}$ remains relatively stable (5–50 MPa). Beyond 60°, $E_{1}$ increases markedly, reaching 883 MPa at 90°, corresponding to a rectangular cell configuration where load transfer is predominantly axial. In contrast, smaller angles introduce bending-dominated deformation paths, reducing stiffness. The 176-fold variation across the studied range highlights the critical role of re-entrant angle in tailoring mechanical performance. A threshold at θ = 60° marks the transition from compliant to stiff behavior, offering a geometric design parameter for targeted structural optimization.

The elastic modulus $E_{2}$ decreases progressively as the re-entrant angle increases, primarily due to the underlying load transfer mechanism. Initially, the vertical load is shared by both the ligaments and the inclined struts. However, with a larger re-entrant angle, the load-bearing role of the inclined struts weakens, leading to reduced compressive stiffness and a corresponding decline in $E_{2}$. In contrast, the shear modulus $G_{12}$ exhibits a non-monotonic trend—rising at first and then declining—with changes in the re-entrant angle, though the fluctuation remains modest.

The maximum $G_{12}$ value of 4.5 MPa is observed at a re-entrant angle of 80°, while the minimum value of 2.7 MPa occurs at 40°. This trend arises because shear deformation is primarily resisted by the inclined cell struts. A small re-entrant angle weakens the shear resistance of the inclined walls, leading to decreased shear stiffness. As the angle increases, the shear resistance improves correspondingly. However, at a re-entrant angle of 90°—where the honeycomb structure approximates a rectangular configuration—the structure, despite its high axial stiffness, becomes more susceptible to lateral sliding under shear loading, thereby causing a decline in $G_{12}$.

### 6.2. Effect of Strut Slenderness Ratio

Figure 12b illustrates the influence of strut slenderness ($l_{1}/t_{1}$) on the equivalent elastic moduli of the accordion honeycomb. As $l_{1}/t_{1}$ increases from 5 to 30, all moduli ($E_{1}$, $E_{2}$, $G_{12}$) exhibit a decreasing trend. $E_{1}$ declines most significantly, from 690 MPa to 4 MPa, indicating its high sensitivity to slenderness. $G_{12}$ and $E_{2}$ decrease more gradually. The results suggest that slender struts (higher $l_{1}/t_{1}$) promote bending-dominated deformation modes, reducing stiffness and shear resistance. Therefore, for load-bearing applications, lower slenderness ratios ($l_{1}/t_{1}<$ 10) are preferable, while higher ratios may be suitable for flexible, energy-absorbing systems.

### 6.3. Effect of Ligament-to-Strut Length Ratio

Figure 12c shows that increasing $l_{2}/l_{1}$ from 0.6 to 1.1 leads to a decrease in $E_{1}$ (43 MPa → 20 MPa) and $G_{12}$ (5 MPa → 3 MPa), while $E_{2}$ remains largely unaffected. Longer ligaments enhance bending and tensile compliance along the 1-direction but do not significantly affect vertical stiffness. Therefore, minimizing $l_{2}/l_{1}$ (< 0.7) is recommended to maximize in-plane stiffness ($E_{1}$), whereas ligament length can be adjusted without compromising vertical load-carrying capacity. This parametric study emphasizes the decoupled roles of ligaments in directional stiffness, offering a geometric strategy to independently tune $E_{1}$ and $G_{12}$ without affecting $E_{2}$.

### 6.4. Effect of Ligament Slenderness Ratio

Figure 12d illustrates the variation in the equivalent elastic moduli of the accordion honeycomb structure as the ligament slenderness ratio $l_{2}/t_{2}$ increases from 4.17 to 25. As $l_{2}/t_{2}$ increases, all moduli—$E_{1}$, $E_{2}$, and $G_{12}$ —exhibit a decreasing trend. Specifically, $E_{1}$ decreases from 39.6 MPa at $l_{2}/t_{2}$ =4.17 to 34.5 MPa at $l_{2}/t_{2}$=25. This relatively minor reduction indicates that changes in the ligament slenderness ratio have limited influence on axial stiffness along the 1-direction, as the overall cell shape and load transfer mechanisms remain largely unchanged. In contrast, $G_{12}$ decreases linearly from 5.4 MPa to 2.2 MPa, representing a reduction of more than twofold. This pronounced decline is attributed to the weakened lateral connectivity between adjacent cells as $l_{2}/t_{2}$ increases, which facilitates intercellular sliding and reduces overall shear resistance. The elastic modulus $E_{2}$ exhibits the most significant decrease, dropping from 32.5 MPa to 6.5 MPa—a reduction of approximately 400%. This substantial decline arises because increasing $l_{2}/t_{2}$ leads to a more slender cellular geometry, making the structure increasingly sustainable to buckling failure under compression.

To support design applications, Table 10 summarizes the preferred parameter ranges for achieving key mechanical performance objectives based on the parametric trends observed.

## 7. Comparison of Different Honeycomb Structures with Zero Poisson’s Ratio

**Figure 13:**
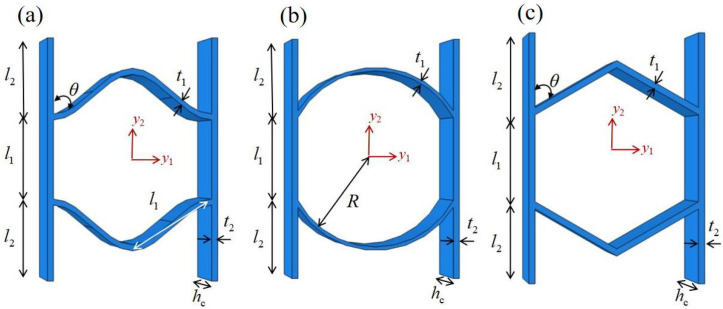
Schematic diagrams of unit cells for three ZPR honeycomb cells: (**a**) sinusoidal, (**b**) circular, and (**c**) accordion designs.

To further evaluate the mechanical performance of zero Poisson’s ratio (ZPR) honeycomb structures, a comparative analysis of the equivalent specific moduli was conducted across three distinct designs: sinusoidal, circular, and accordion honeycombs. To ensure comparability, all structures were fabricated with identical length, width, and height dimensions, and aluminum alloy was selected as the base material. The unit cell configurations are illustrated in Figure 13, where the ZPR-sinusoidal cell follows a wave function y=3×sin(0.24x), the ZPR-circular cell consists of circular arcs with a radius of 15 mm, and the ZPR–accordion cell adopts an inclined strut arrangement.

**Figure 14:**
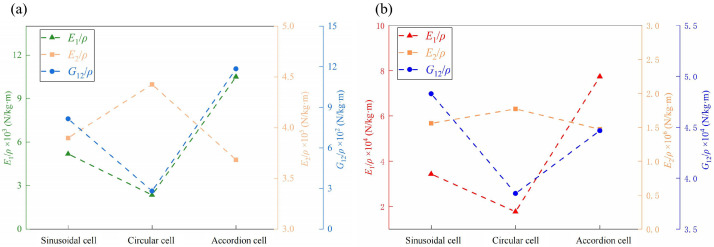
Comparison of equivalent specific modulus for different honeycomb structures with zero Poisson’s ratio: (**a**) in-plane and (**b**) out-of-plane properties.

Figure 14 compares the specific elastic moduli in both in-plane and out-of-plane directions across three ZPR-honeycomb configurations. In terms of in-plane properties (Figure 14a), the ZPR–accordion honeycomb exhibits the highest specific elastic modulus $E_{1}/\rho \left(1.05\times 10^{4}\;N/\mathrm{kg}\cdot m\right)$ and specific shear modulus $G_{12}/\rho \left(1.2\times 10^{3}\;N/\mathrm{kg}\cdot m\right)$, indicating superior stiffness along the 1-direction (horizontal axis). This enhanced performance is attributed to its straight inclined struts, which efficiently resist axial and shear loads. In contrast, the ZPR-circular honeycomb shows the lowest $E_{1}/\rho \left(2.355\times 10^{3}\;N/\mathrm{kg}\cdot m\right)$ and $G_{12}/\rho \left(2.8\times 10^{2}\;N/\mathrm{kg}\cdot m\right)$, although it achieves the highest $E_{2}/\rho \left(4.4\times 10^{5}\;N/\mathrm{kg}\cdot m\right)$ due to its anisotropic geometry, which promotes uniform load distribution along the 2-direction (vertical axis). The ZPR-sinusoidal honeycomb exhibits intermediate performance, as its curved struts are more susceptible to bending deformation, resulting in lower stiffness compared to the ZPR–accordion honeycomb.

For out-of-plane properties (Figure 14b), the ZPR–accordion honeycomb again achieves the highest out-of-plane specific elastic modulus $E_{1}/\rho \left(7.74\times 10^{4}\;N\cdot {\mathrm{kg}}^{-1}\cdot m^{-1}\right)$, outperforming the ZPR-sinusoidal and ZPR-circular designs by factors of 2.3 and 4.4, respectively. This superior performance stems from the straight strut configuration, which enhances bending resistance along the 1-direction. In terms of out-of-plane properties, the differences in $E_{2}/\rho$ among the three designs are relatively minor, with the ZPR-circular honeycomb achieving the highest value, followed by the ZPR-sinusoidal and ZPR-honeycombs. This trend indicates that curved struts facilitate better stress redistribution and deformation resistance along the 2-direction.

The comparison among ZPR topologies is based on FEM simulations. Experimental validation was conducted only for the accordion-type structure. However, the simulation results demonstrate that the ZPR–accordion honeycomb offers the best in-plane and out-of-plane stiffness along the primary loading axis, making it a promising candidate for applications requiring high specific modulus and dimensional stability. The ZPR-sinusoidal honeycomb, characterized by pronounced bending flexibility, is more suitable for applications demanding higher compliance, while the ZPR-circular honeycomb excels in providing anisotropic mechanical properties and effective load distribution.

It should be noted that the present model and experimental validations are based on millimeter-scale cellular structures (cell size ∼15 mm, relative density 0.163), where classical continuum mechanics remains applicable. For micro- and nanocellular materials, especially those discussed in Le Barbenchon and Kopp [43], additional size effects and surface energy contributions may alter the scaling laws and enhance mechanical performance. Extending the current model to such regimes would require the incorporation of nonlocal or gradient effects, which is beyond the scope of this work.

## 8. Conclusions

In this study, a comprehensive analytical approach, combining three-dimensional equivalent Cauchy continuum modeling (3D-ECM), finite element simulations (3D-FEM), and experimental validation through quasi-static compression tests, has been employed to investigate the mechanical performance of the ZPR–accordion honeycomb.

The results confirm that the ZPR–accordion honeycomb exhibits zero lateral deformation during compression, maintaining dimensional stability while absorbing significant energy. Specifically, the 3D-ECM, developed using the variational asymptotic method (VAM), has shown high predictive accuracy when compared with experimental results, with deviations under 10%, validating its effectiveness as a computational tool for large-scale simulations and structural optimization.

Parametric studies indicate that key geometric parameters—such as re-entrant angle, strut slenderness ratio, ligament-to-strut length ratio, and ligament slenderness ratio—significantly affect the equivalent elastic moduli and shear modulus. The results suggest that careful tuning of these parameters can optimize the mechanical performance of the accordion honeycomb for specific applications. For instance, increasing the re-entrant angle enhances axial stiffness, while reducing the ligament slenderness ratio can improve shear resistance.

Comparative analysis of different Poisson’s ratio honeycombs (hexagonal, re-entrant, and accordion) reveals distinct performance characteristics. The re-entrant honeycomb exhibits superior energy-absorption efficiency, whereas the hexagonal honeycomb excels in load redistribution, leading to a longer plateau stage. However, the accordion honeycomb, while demonstrating lower energy-absorption efficiency, offers unmatched stability and minimal lateral expansion, making it ideal for applications requiring high dimensional stability under compression.

While this study focuses on the mechanical modeling and experimental validation of the accordion honeycomb core, we acknowledge that the complete structural performance of sandwich composites—comprising both the core and face sheets—is critical for practical applications. Future work will aim to extend the proposed VAM-based equivalent continuum model to analyze full sandwich panels under various loading conditions, such as bending, shear, and impact. This extension can be achieved by incorporating the homogenized core properties into global plate or shell theories, as demonstrated in previous studies [44,45]. The current work thus lays the theoretical foundation for multiscale modeling and optimization of lightweight sandwich structures incorporating zero Poisson’s ratio cores.

## Figures and Tables

**Table 1:** Cellular geometric parameters of honeycomb structures (unit: mm).

Parameters	$l_{1}$	$l_{2}$	$t_{1}$	$t_{2}$	*h*	$\theta \;{(}^{\circ })$
Values	15	12.5	1	1	10	60

**Table 2:** Equivalent engineering constants for ZPR–accordion honeycomb obtained from VAM-based homogenization.

Items	$E_{1}$ (MPa)	$E_{2}$ (MPa)	$E_{3}$ (MPa)	$G_{12}$ (MPa)	$G_{13}$ (MPa)	$G_{23}$ (MPa)	$\nu_{12}$	$\nu_{13}$	$\nu_{23}$	ρ (g/cm^3^)
Values	180.36	7.81	287.57	0.591	60.674	38.859	−0.1065	0.2444	0.0106	0.163

**Table 3:** Quasi-static compression deformation process of honeycomb structures with different Poisson’s ratios.

Strain	0	0.1	0.2	0.3	0.4
Accordion	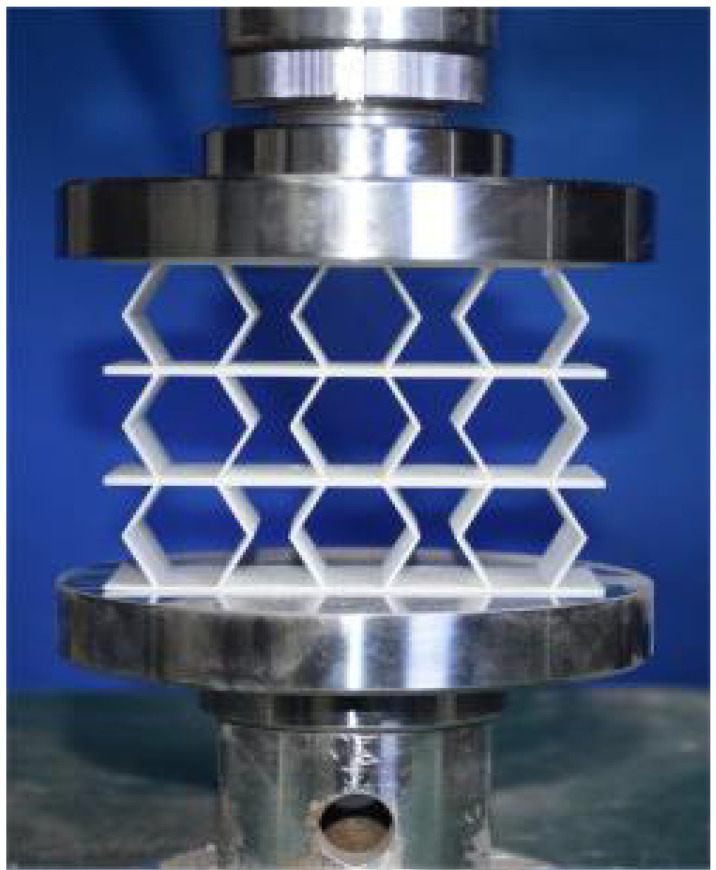	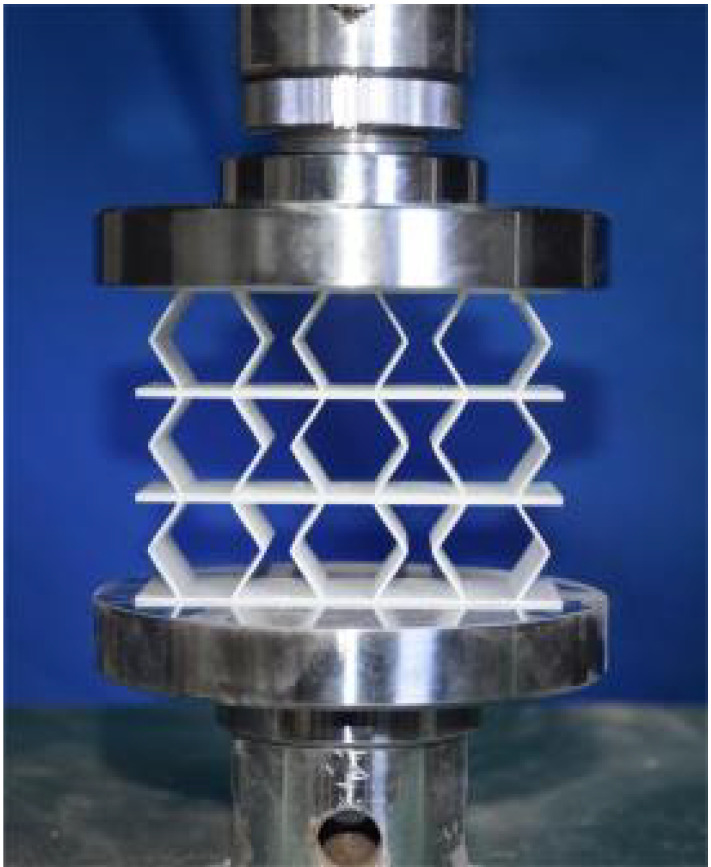	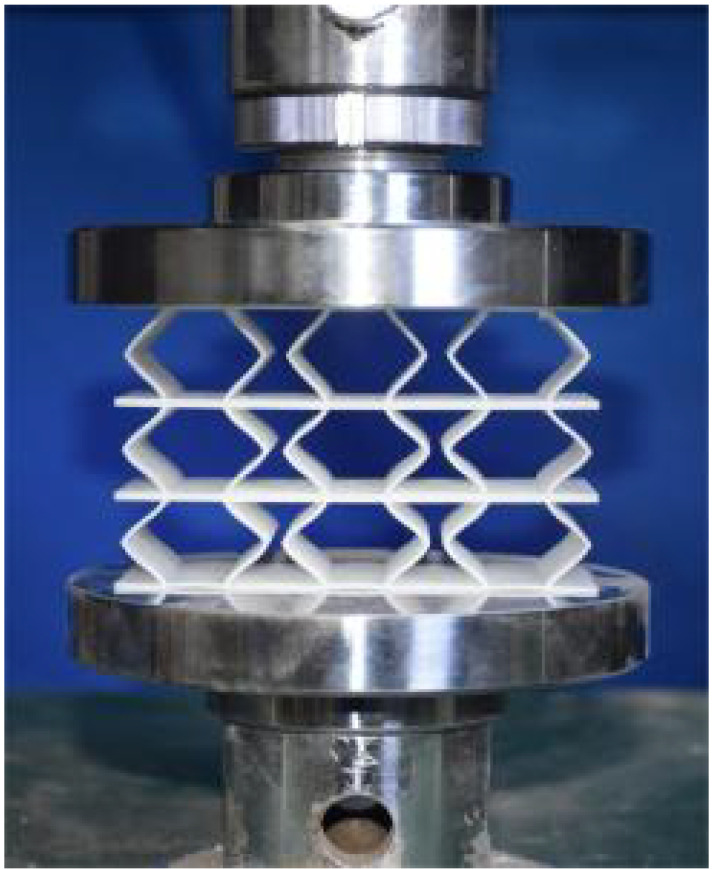	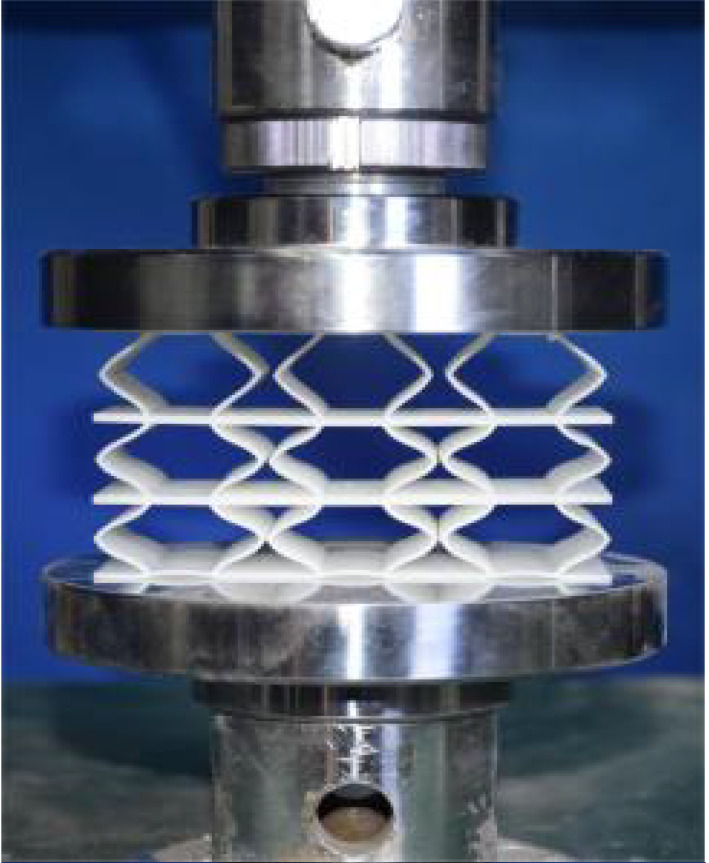	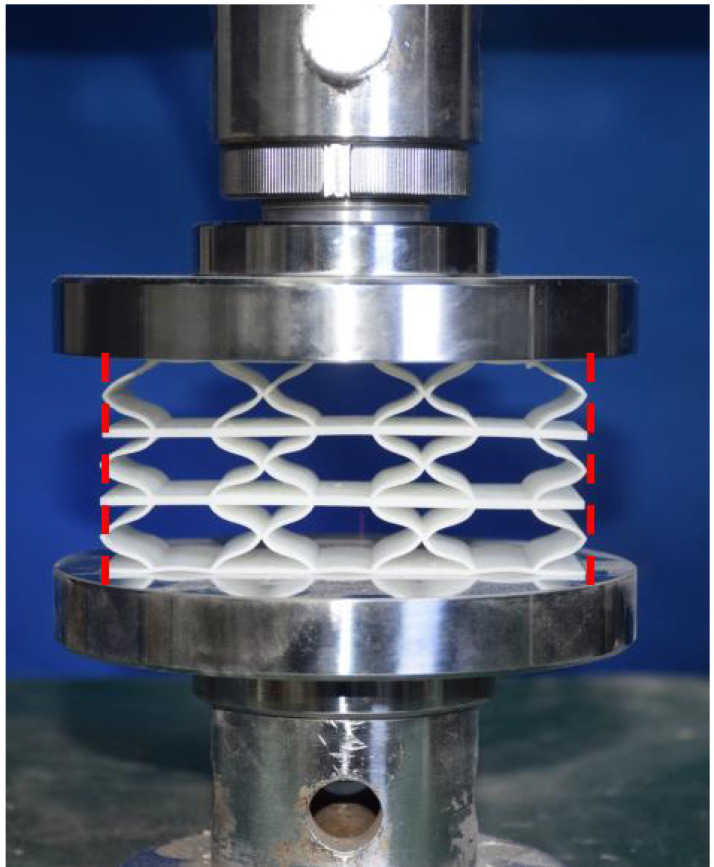
Re-entrant	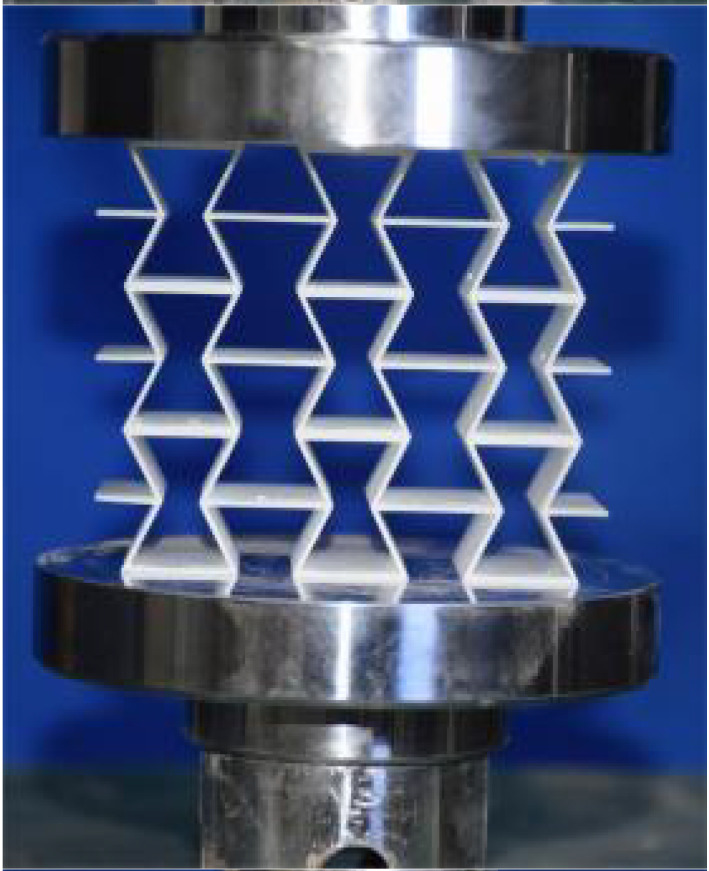	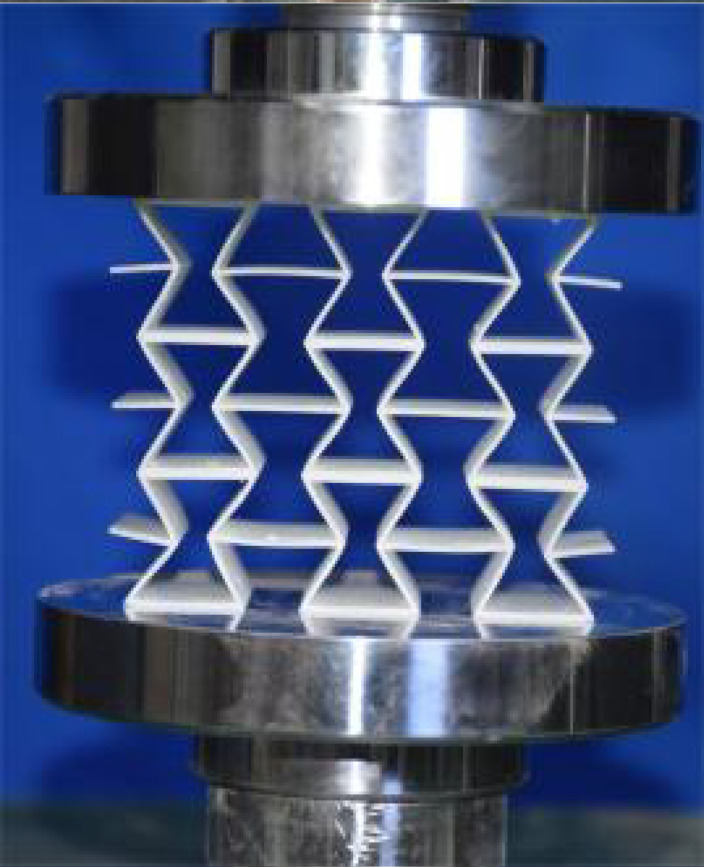	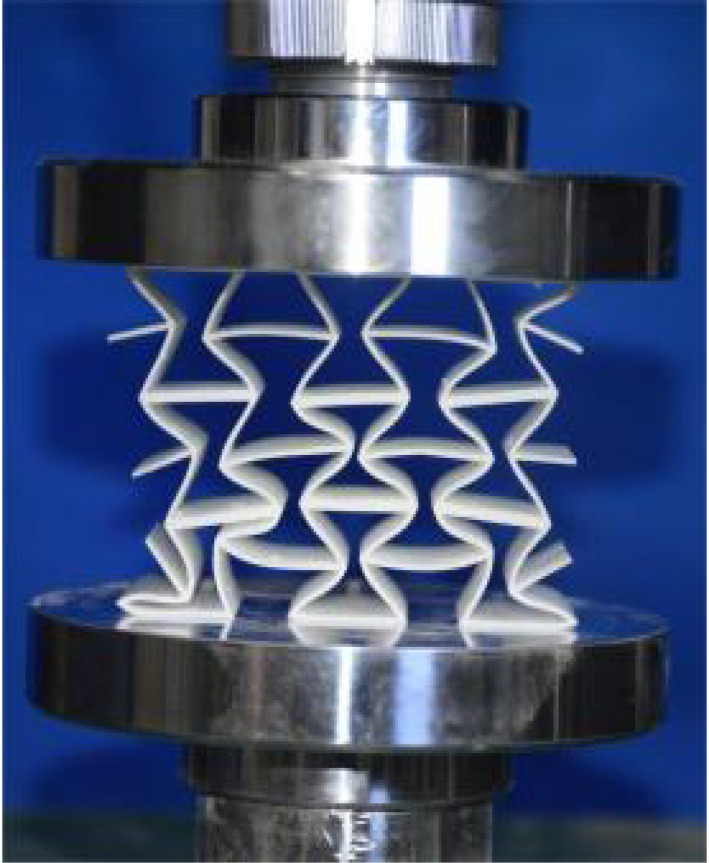	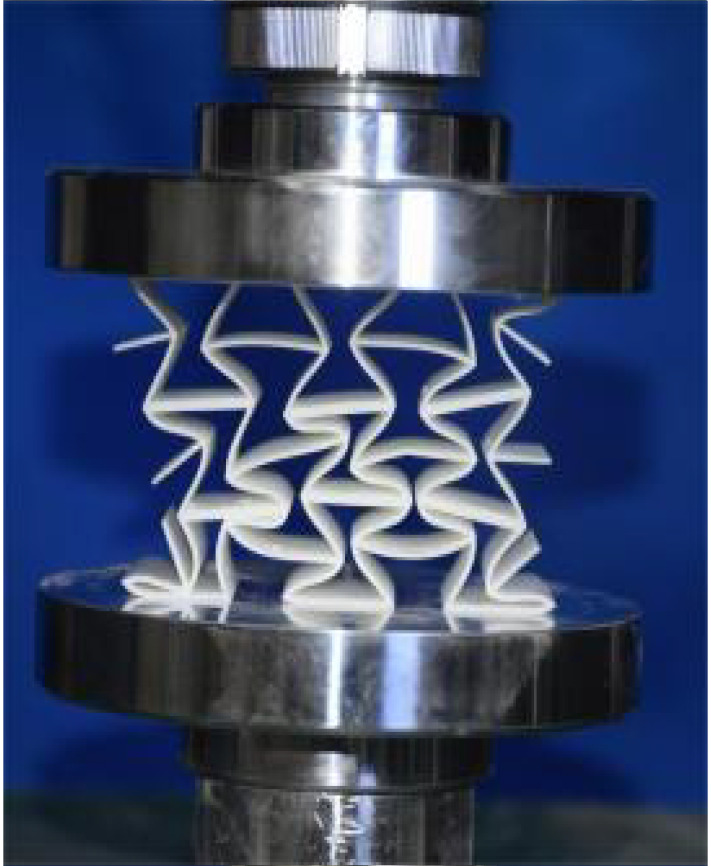	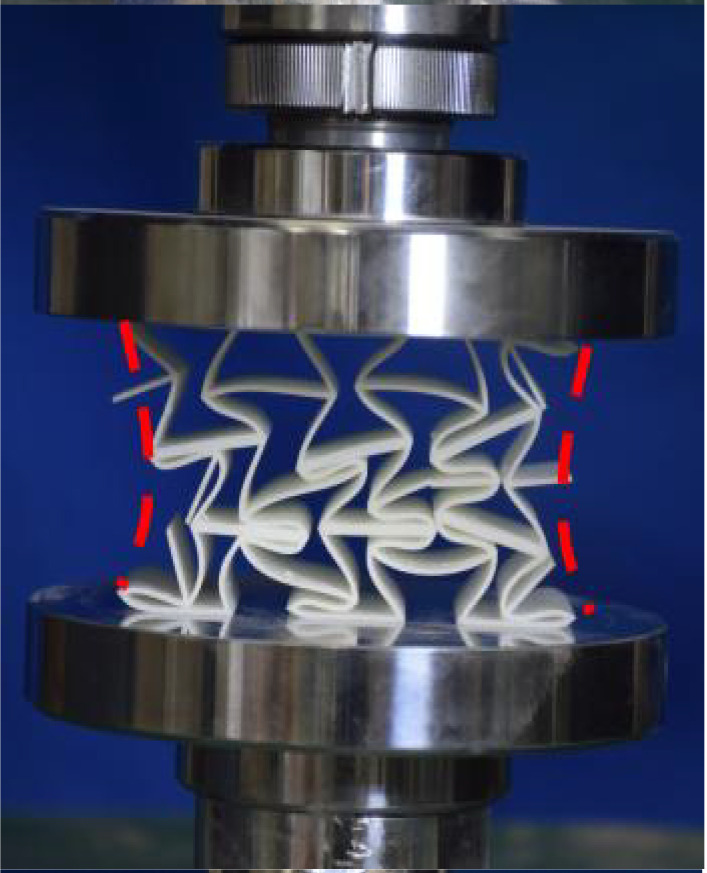
Hexagon	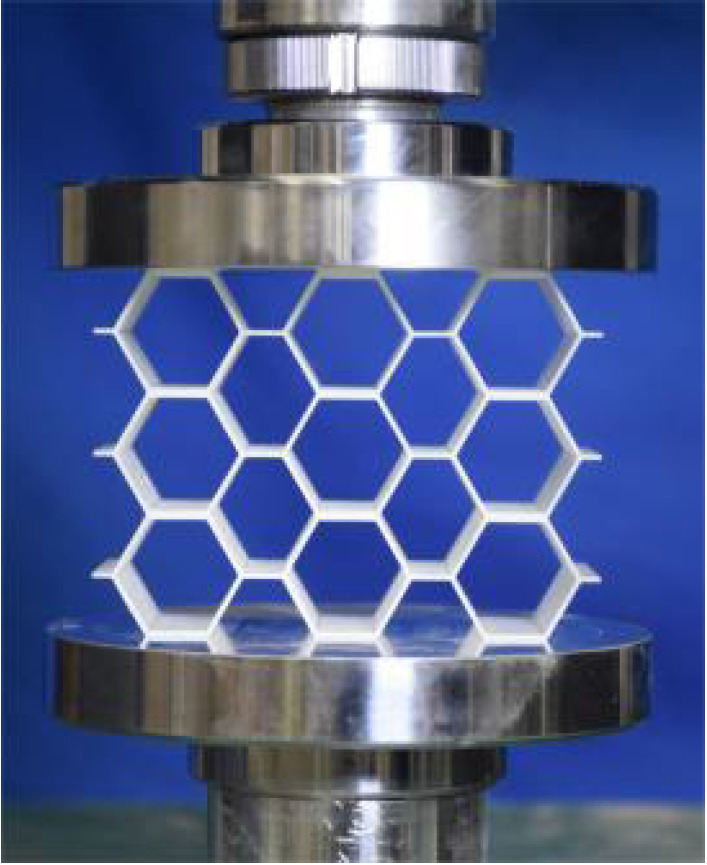	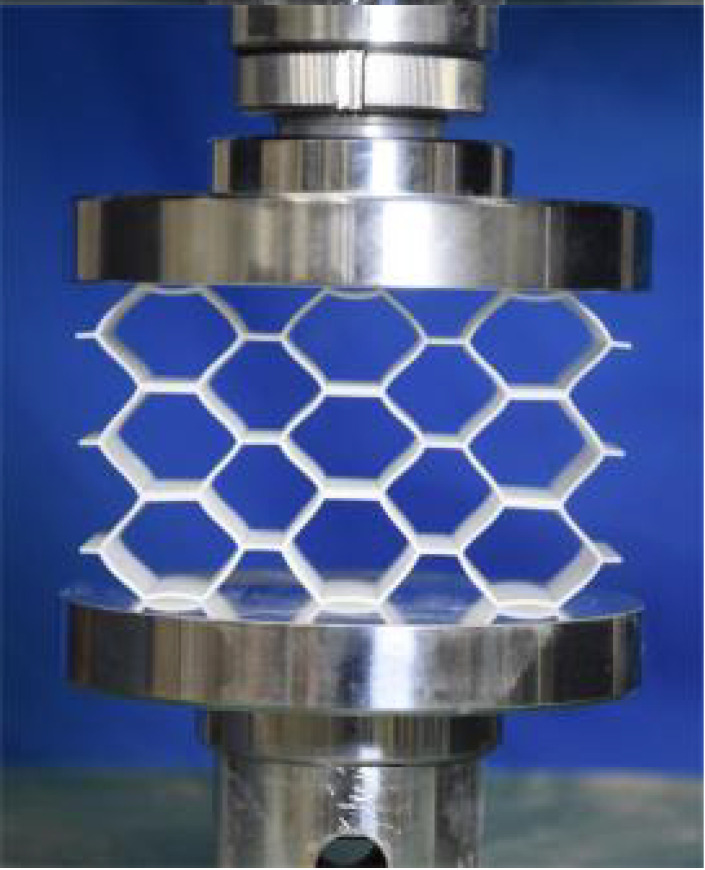	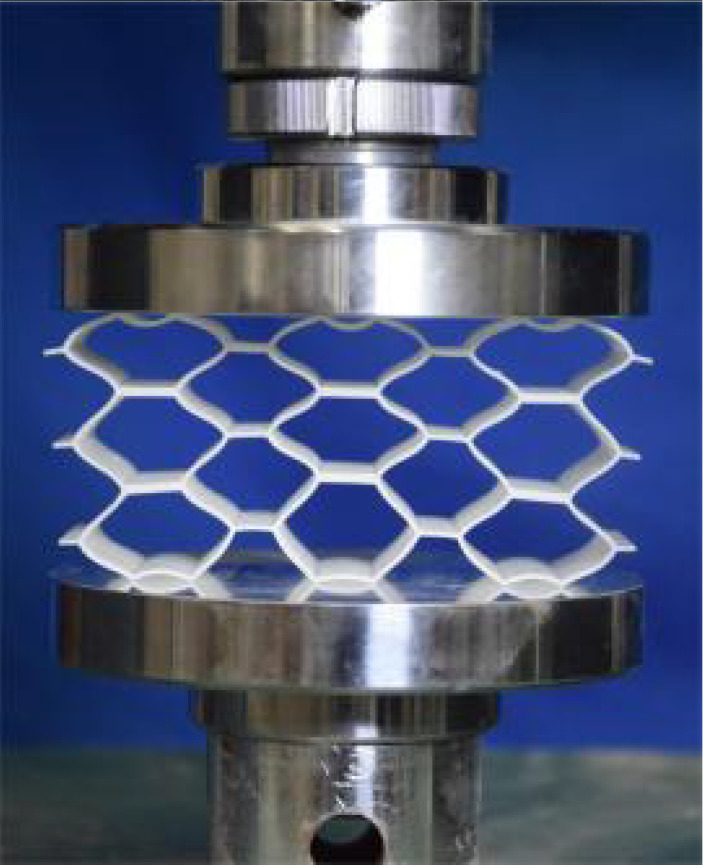	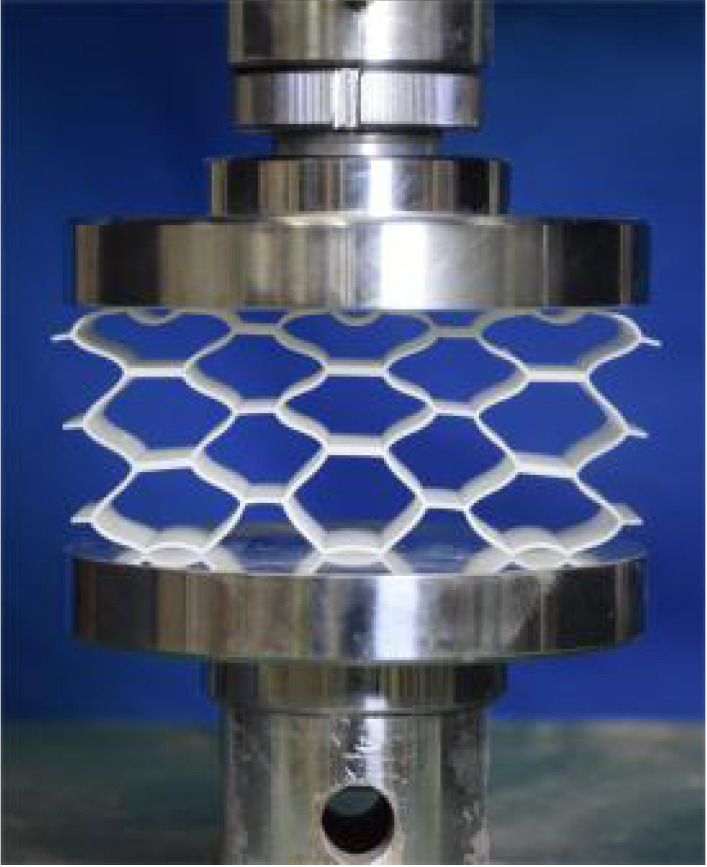	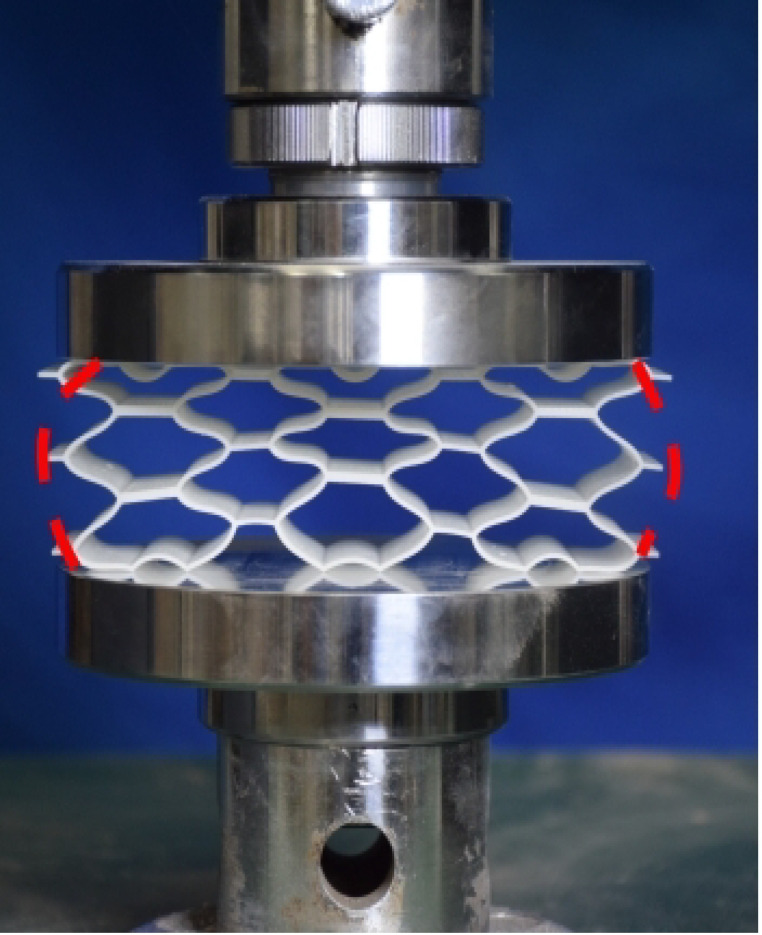

**Table 4:** Summary of onset strain of plateau stage ($\varepsilon_{o}$) and densification strain ($\varepsilon_{d}$) for each structure.

Structure	$\varepsilon_{o}$	$\varepsilon_{d}$
Accordion honeycomb	0.11	0.25
Hexagon honeycomb	0.16	0.32
Re-entrant honeycomb	0.20	0.30

**Table 5:** Comparison of elastic modulus among experimental (EXP), finite element (FEM), and equivalent Cauchy model (ECM) results (unit: MPa).

Structure	Modulus—EXP	FEM	ECM	FEM Error (%)	ECM Error (%)
Accordion honeycomb	4.85	4.67	4.51	3.7%	7.0%
Hexagon honeycomb	3.53	3.59	3.48	1.7%	7.1%
Re-entrant honeycomb	3.57	3.49	3.46	2.2%	3.1%

**Table 6:** Comparison of predicted displacement distributions under 25 mm compression using 3D-FEM and 3D-ECM.

Models	Re-Entrant Honeycomb	Hexagon Honeycomb	Accordion Honeycomb
3D-FEM	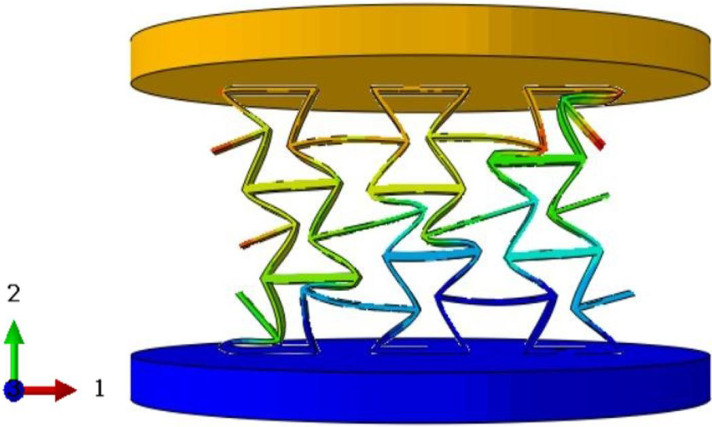	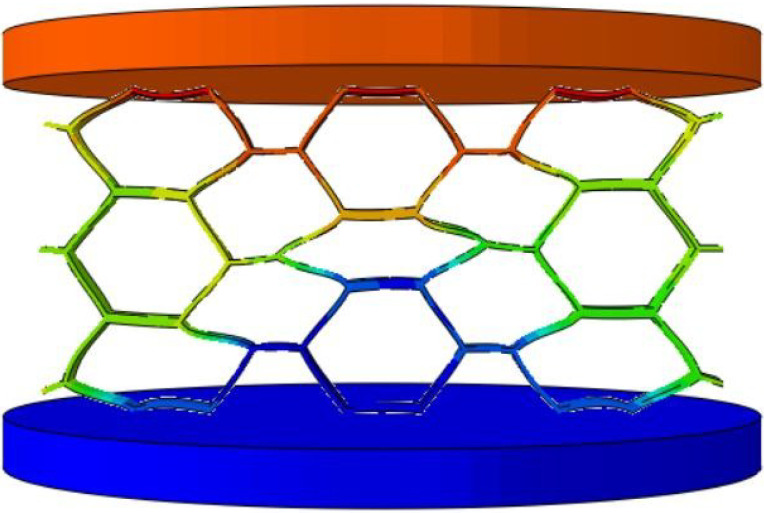	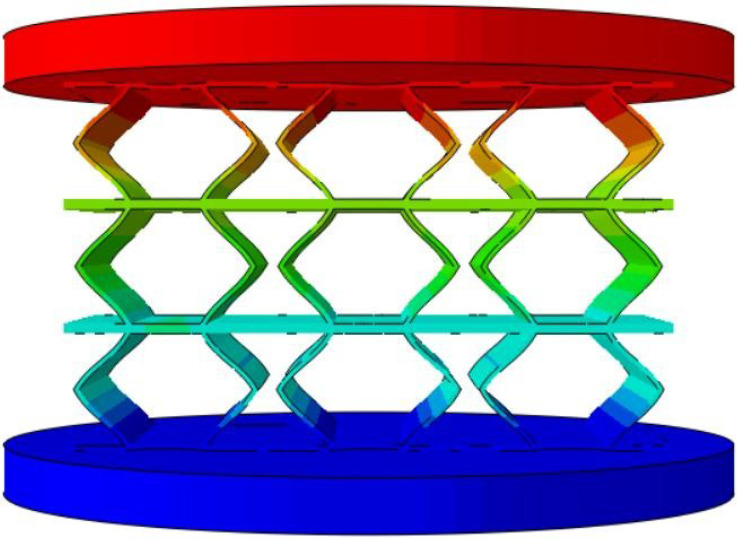
3D-ECM	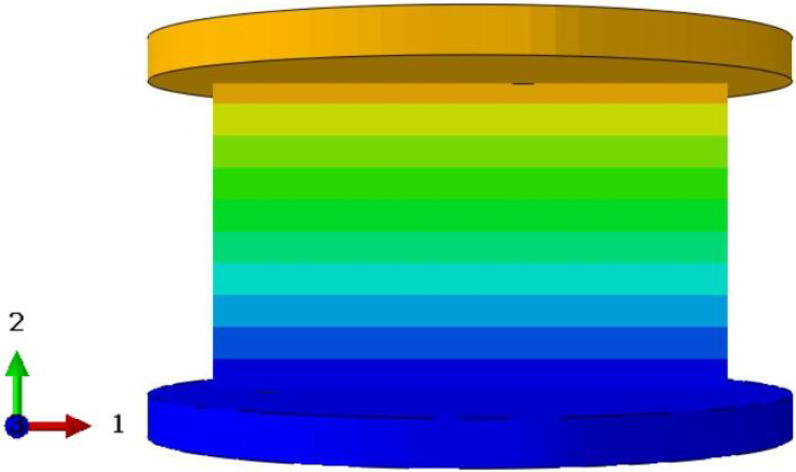	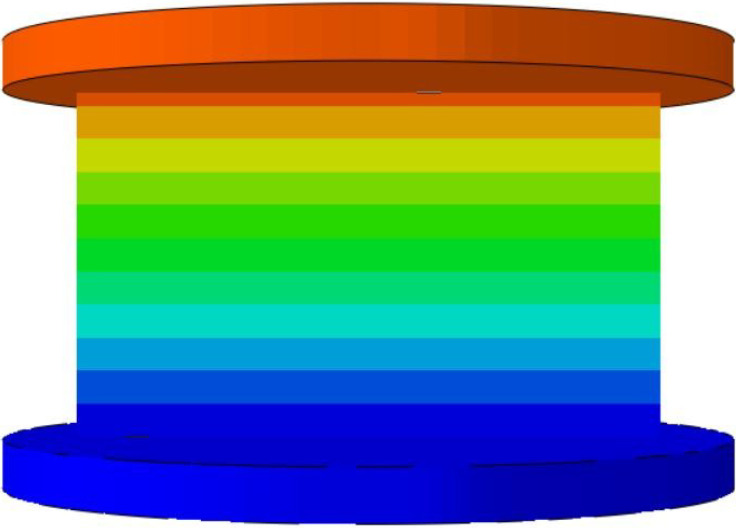	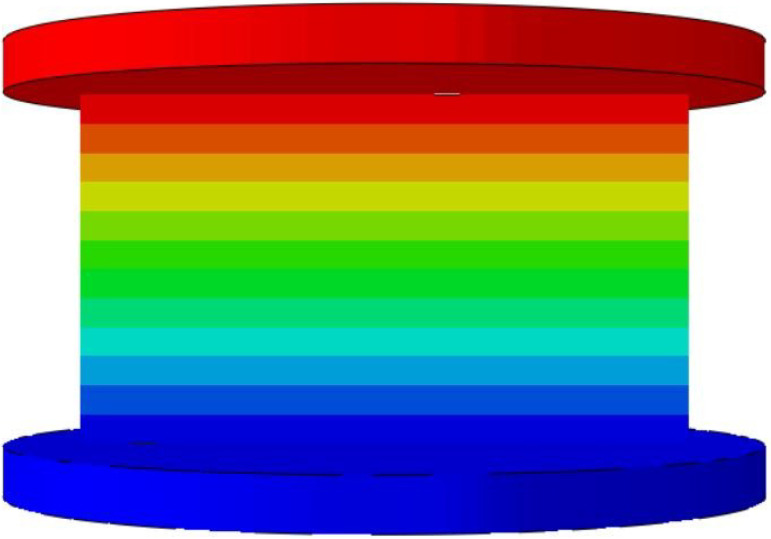

**Table 7:** Comparison of computational efficiency between 3D-ECM and 3D-FEM under quasi-static compression.

Items	Accordion Honeycombs	
	3D-FEM	3D-ECM
Type of elements	C3D10	C3D8R
Number of elements	235,285	77,792
Number of nodes	455,742	83,835
Uniaxial compression	570 s	82 s

**Table 8:** Parameter ranges for structural design of the accordion cell.

$l_{2}/l_{1}$	$l_{1}/t_{1}$	$l_{2}/t_{2}$	$\theta^{\circ }$
0.6∼1.1	5∼30	6.25	60
0.8	15	6.25	60
0.8	15	4.17∼25	60
0.8	15	6.25	40∼90

**Table 9:** Diagram of structural parameters changes in accordion cell (unit: mm).

$\theta^{\circ }$	40	50	60	70	80	90
Cell geometry	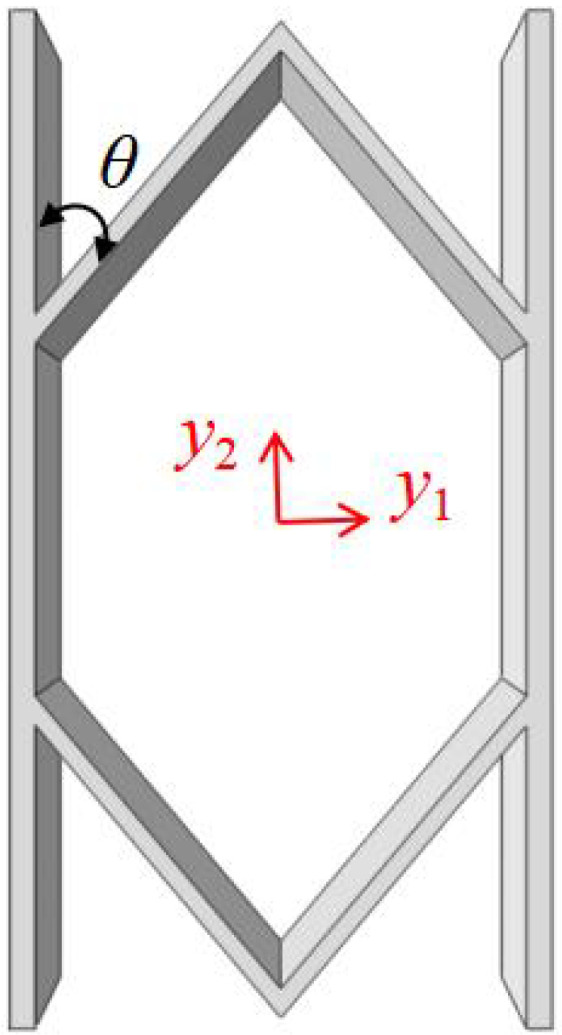	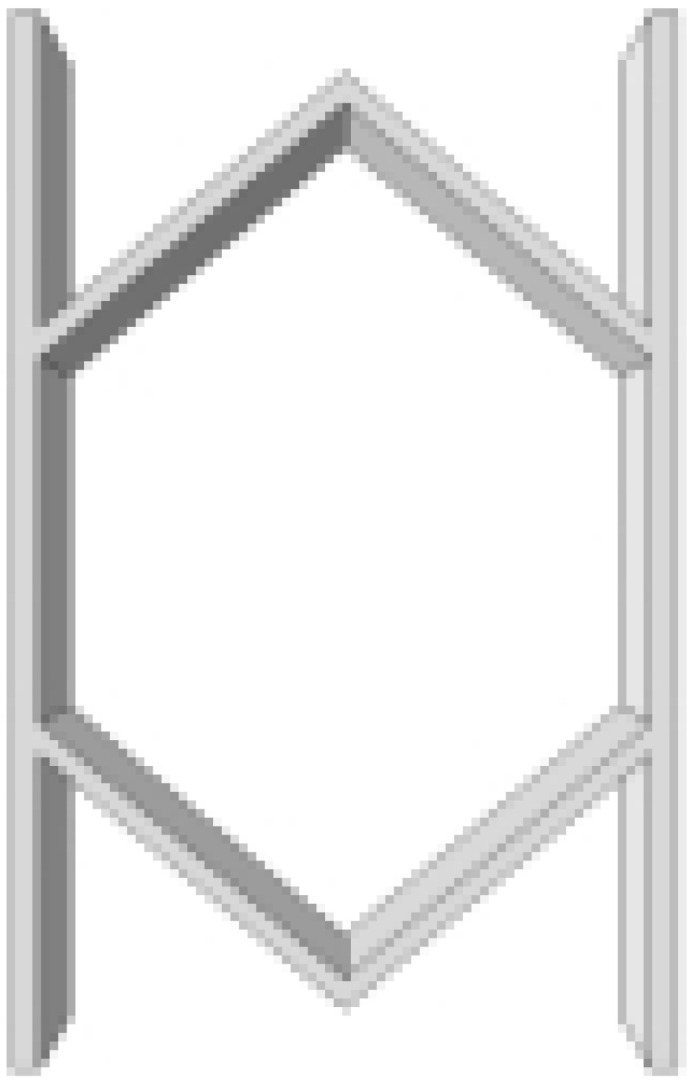	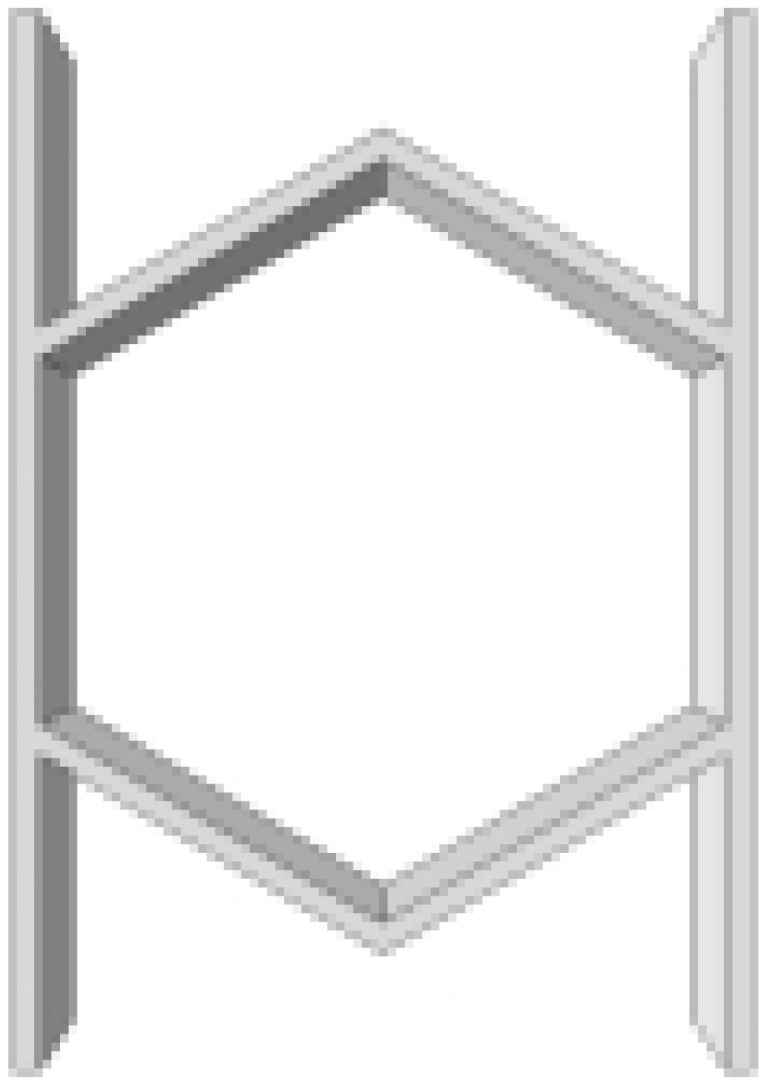	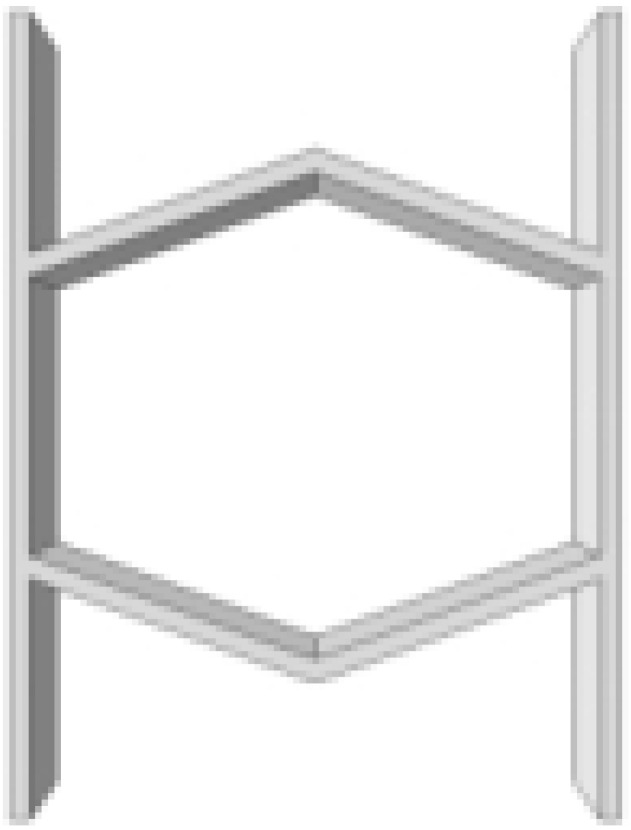	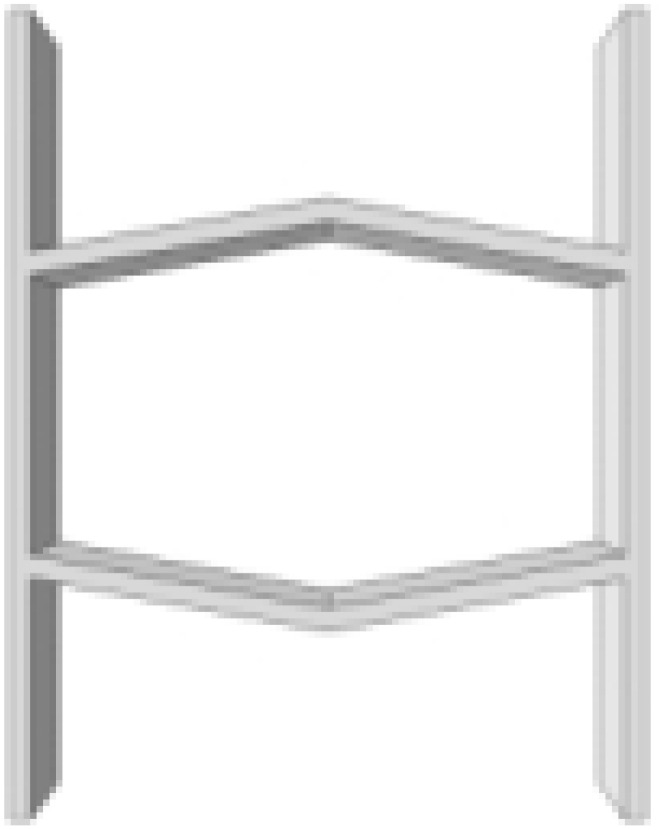	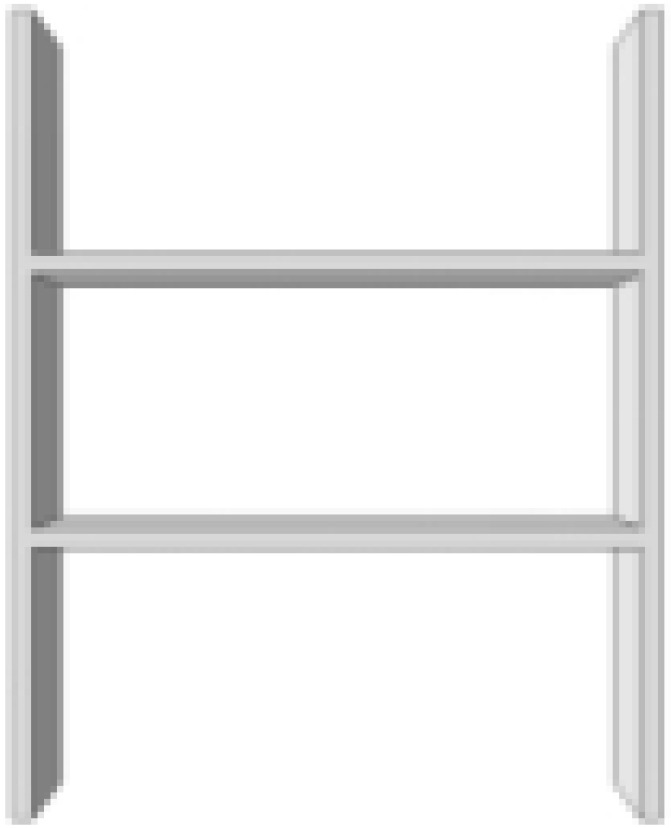
$l_{1}/t_{1}$	5	6	7.5	10	15	30
Cell geometry	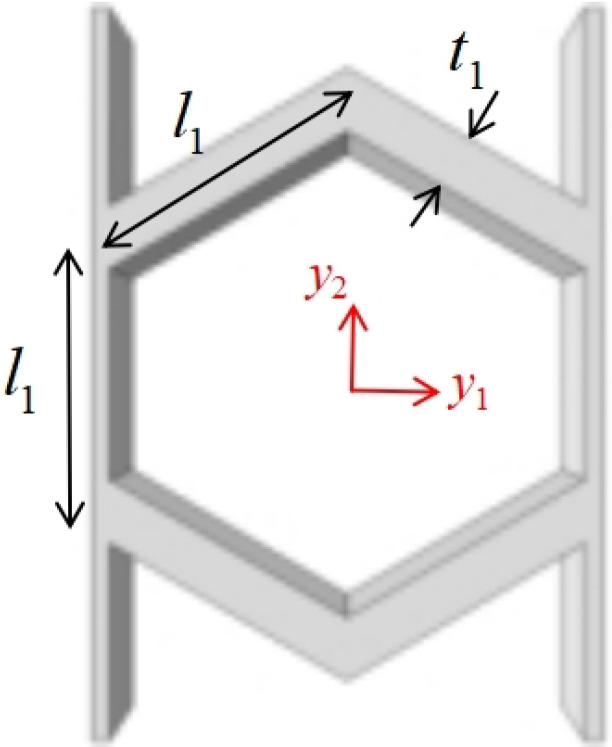	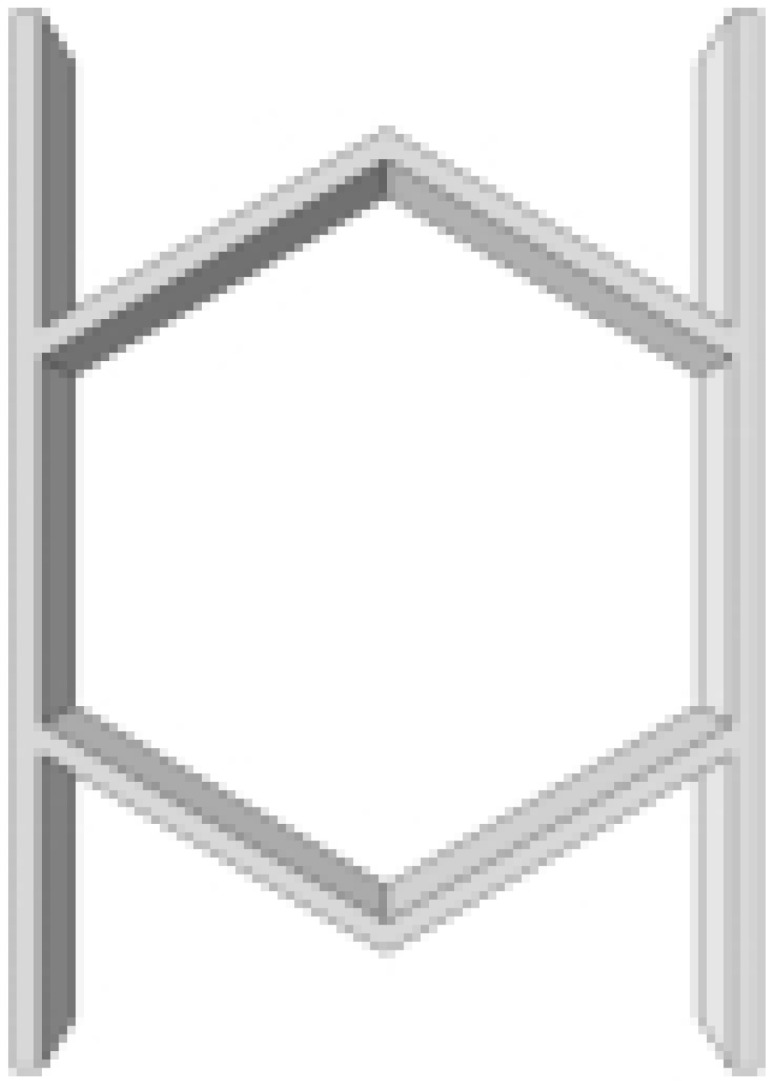	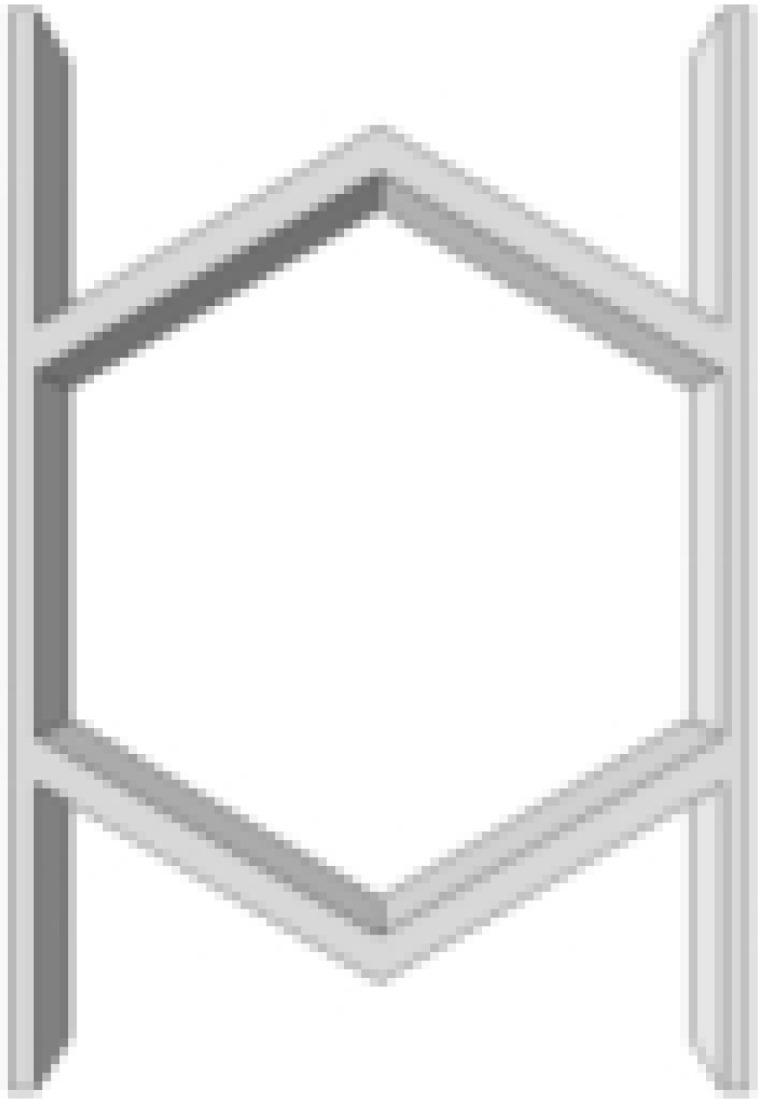	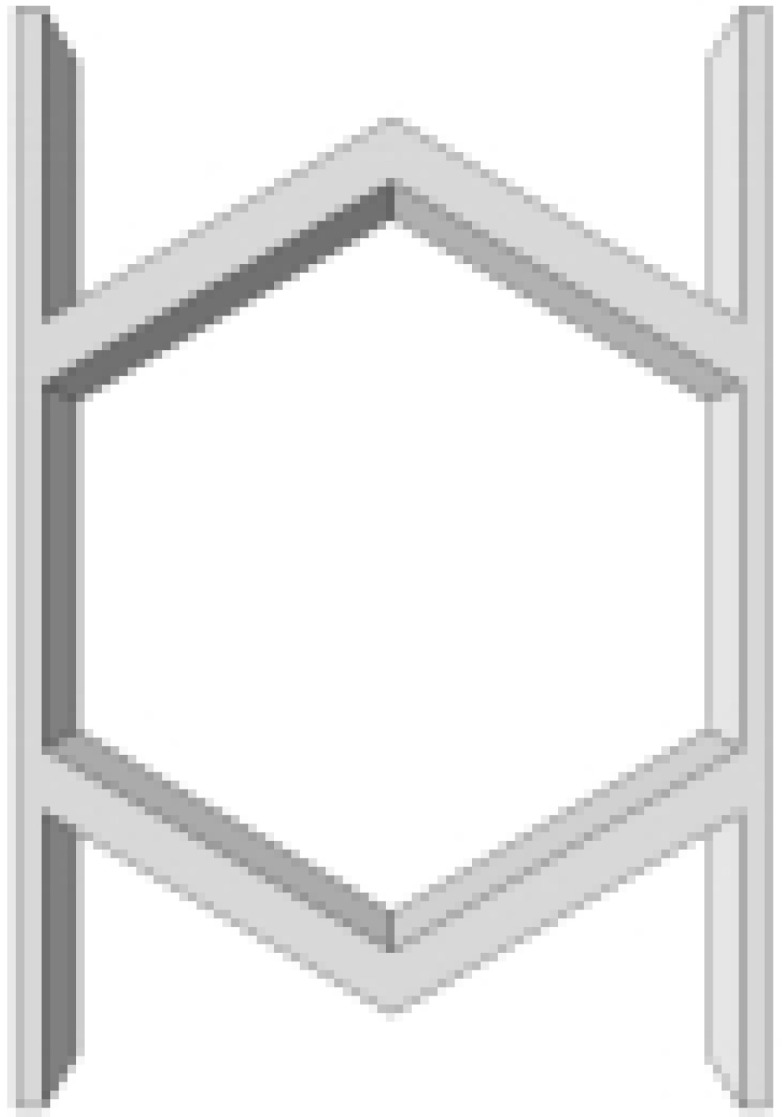	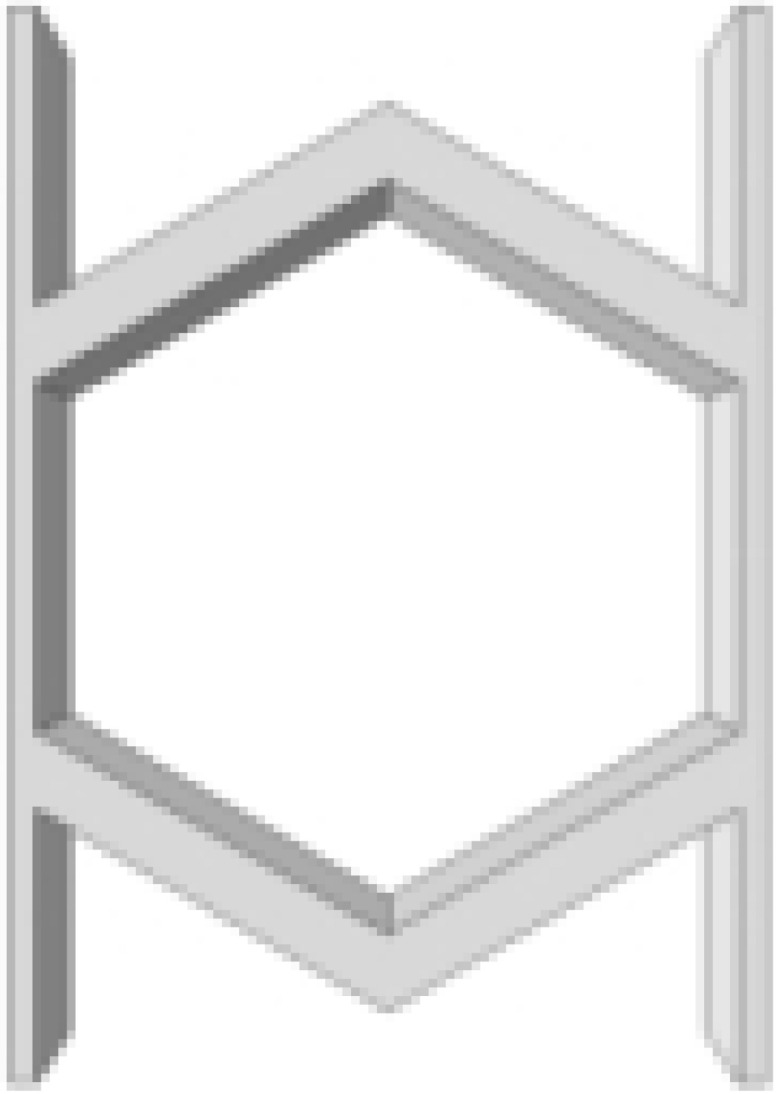	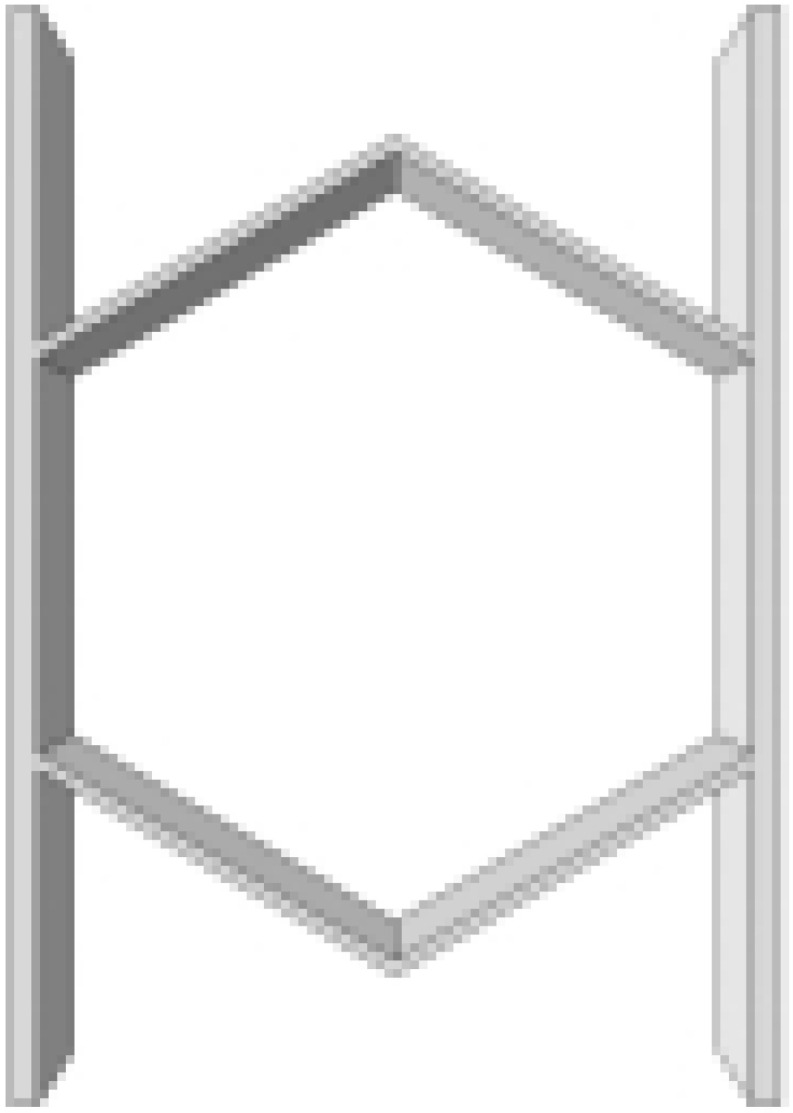
$l_{2}/l_{1}$	0.6	0.7	0.8	0.83	1	1.1
Cell geometry	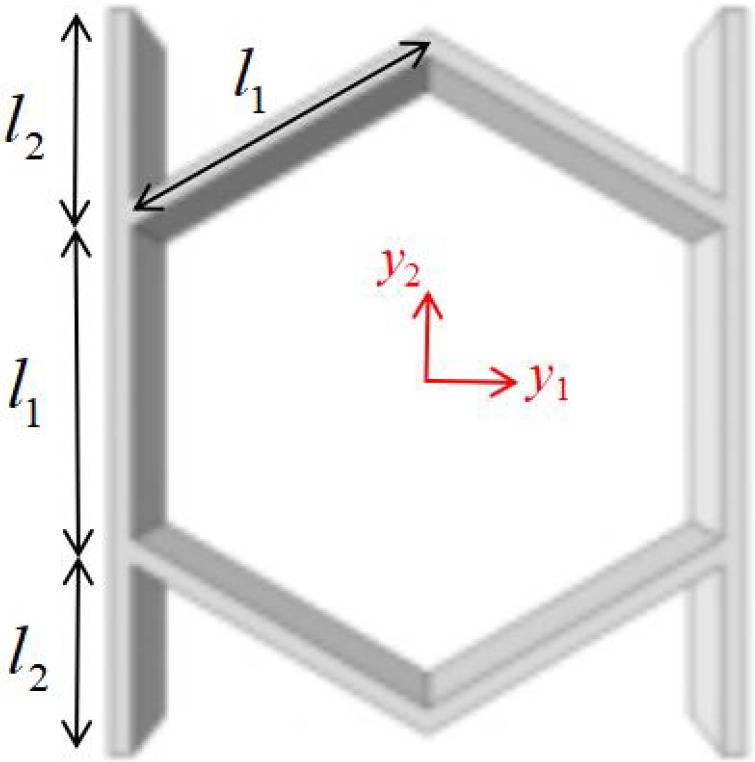	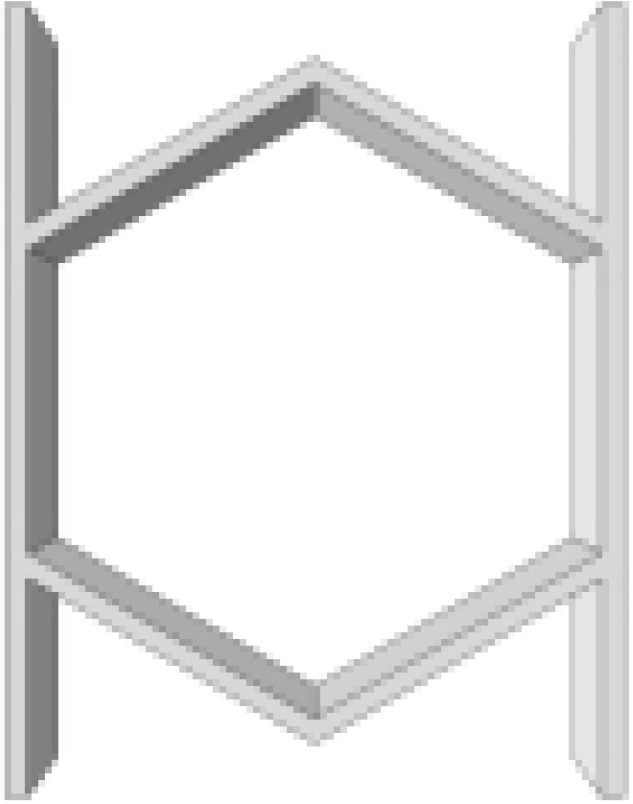	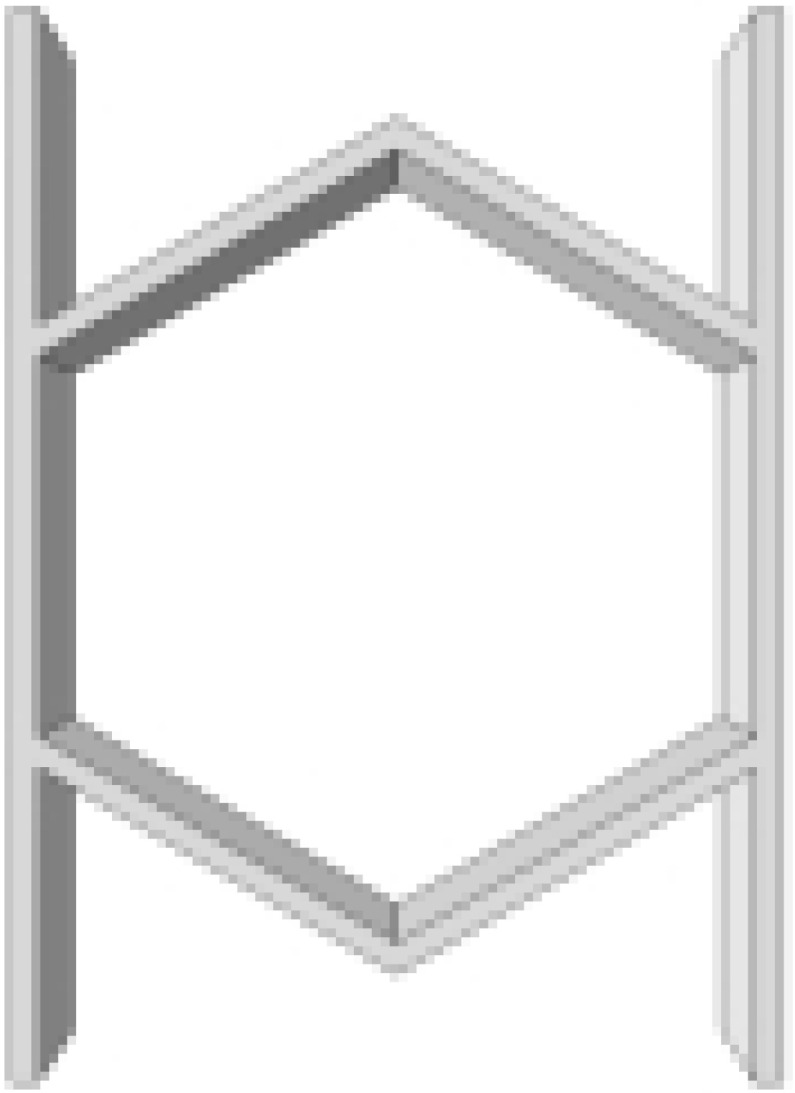	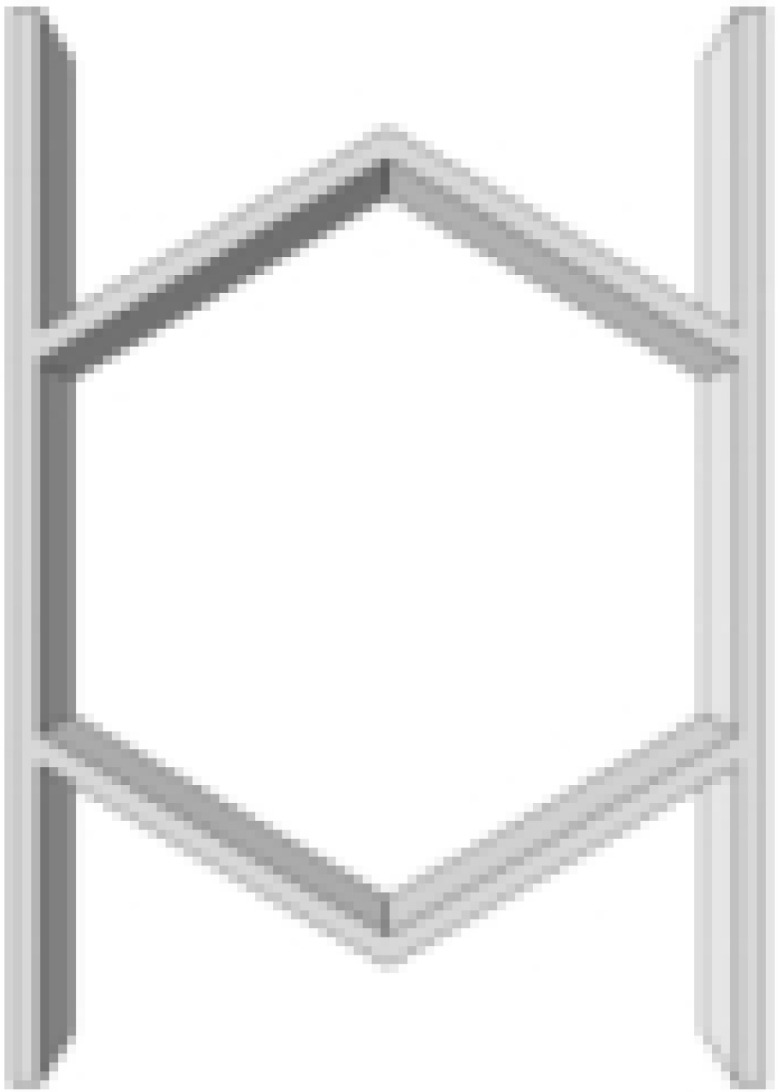	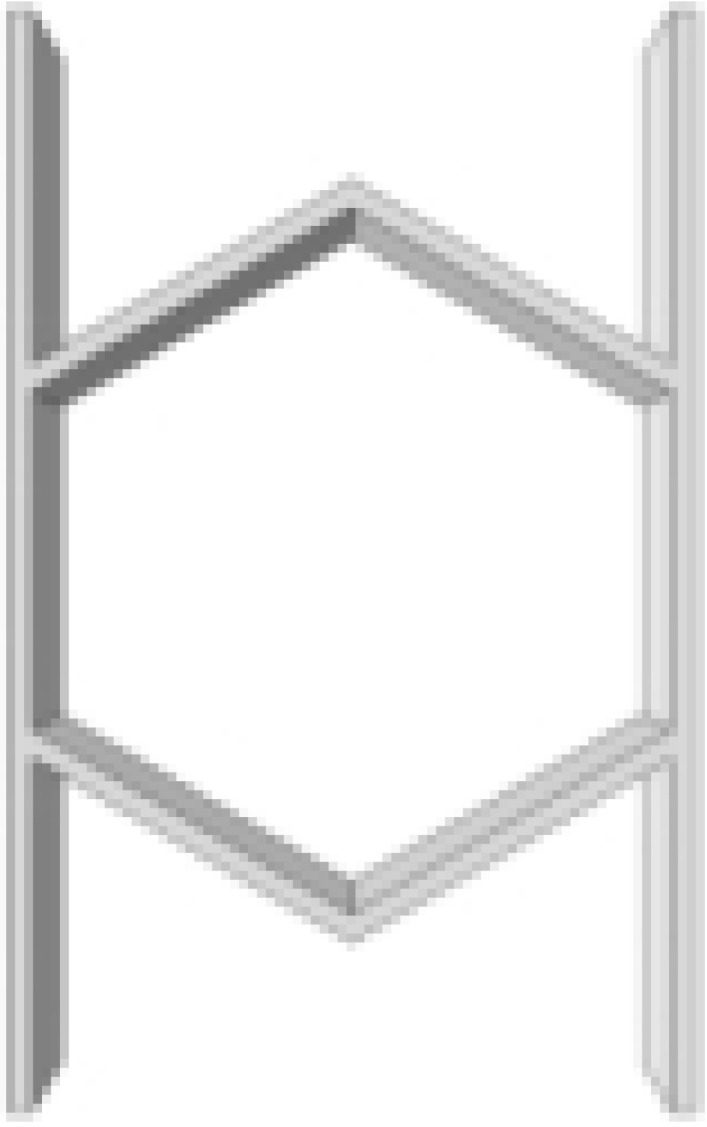	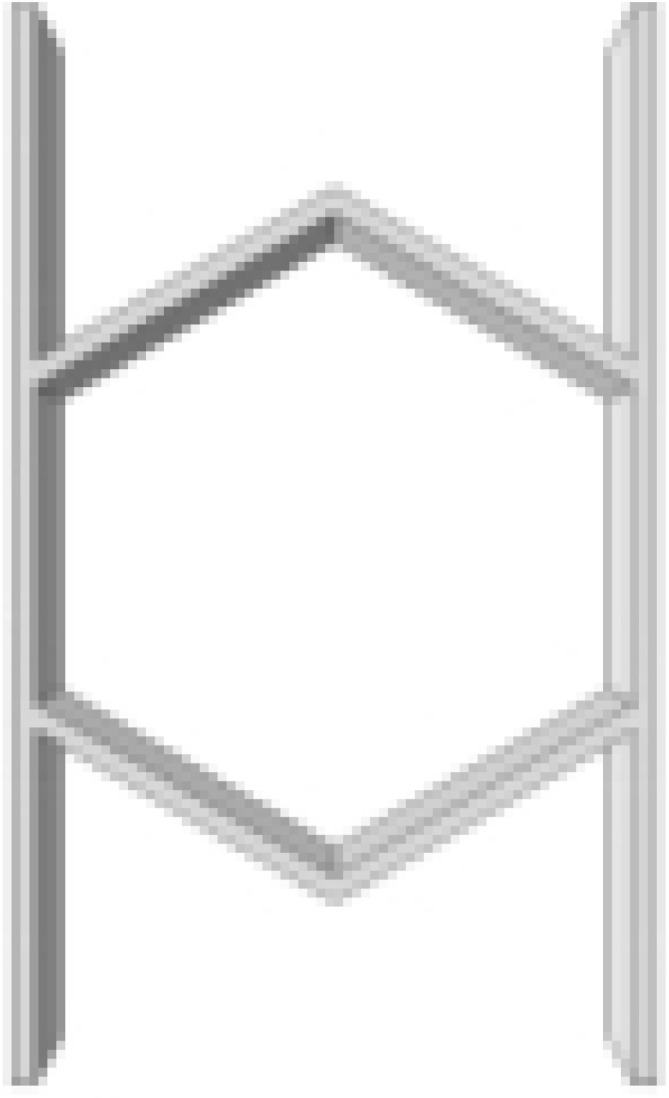
$l_{2}/t_{2}$	4.17	5	6.25	8.33	12.5	25
Cell geometry	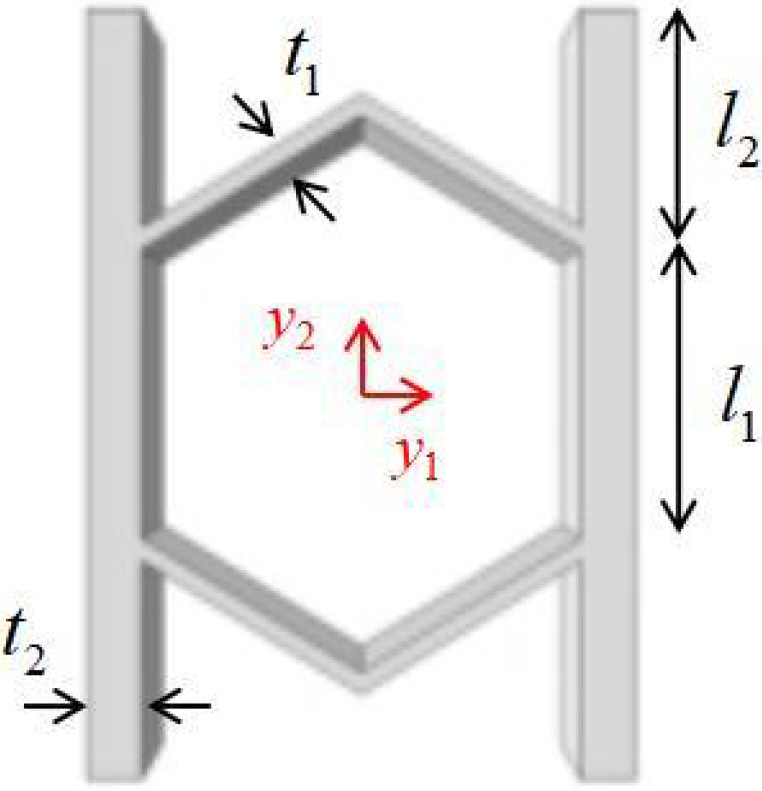	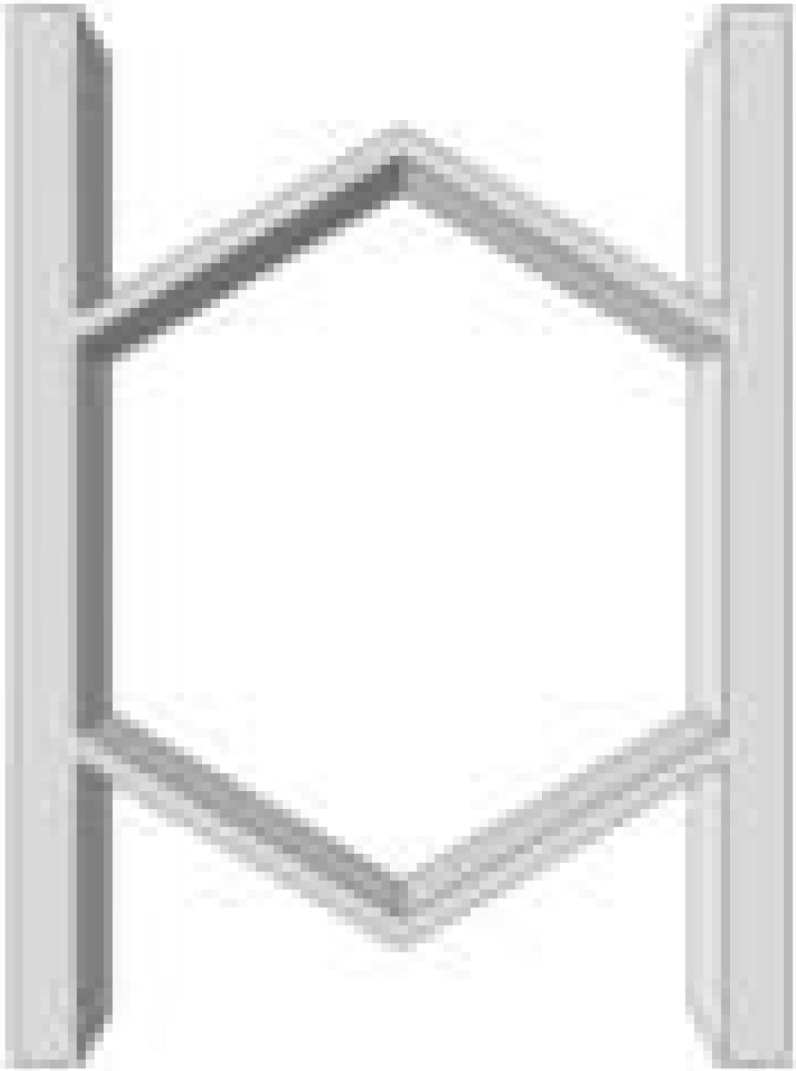	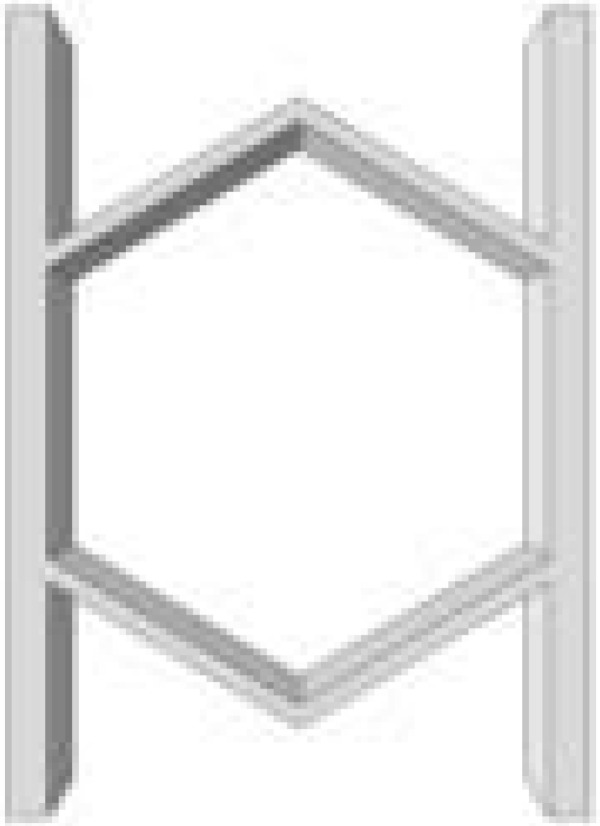	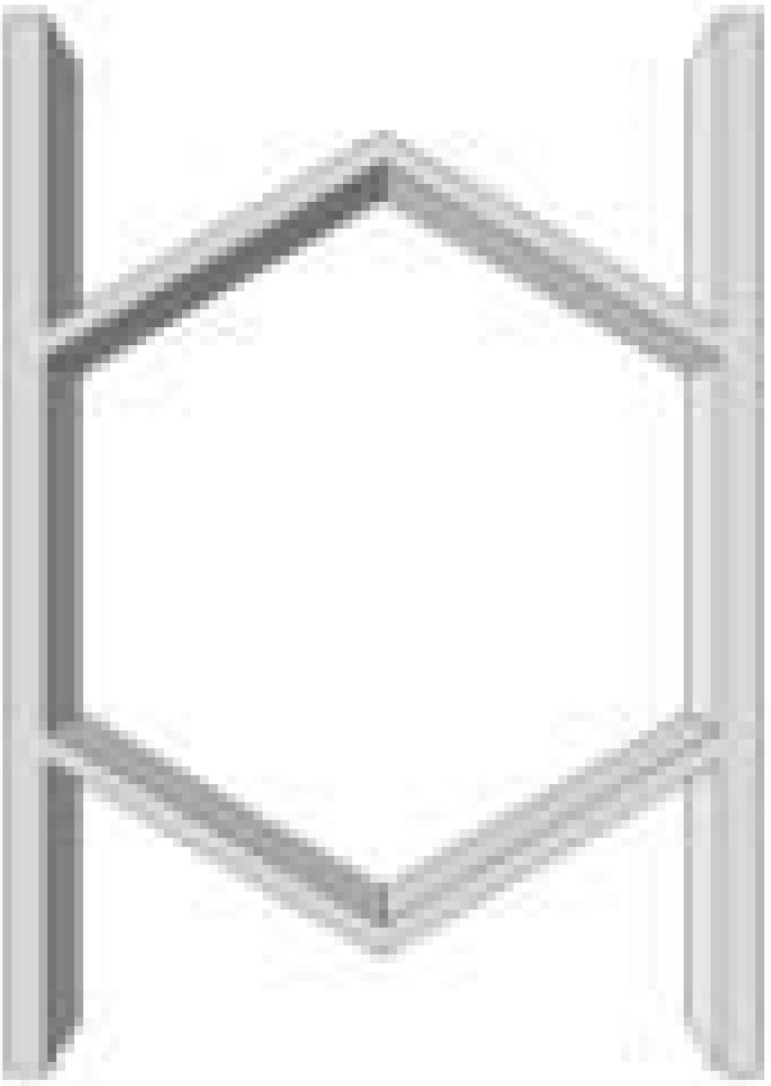	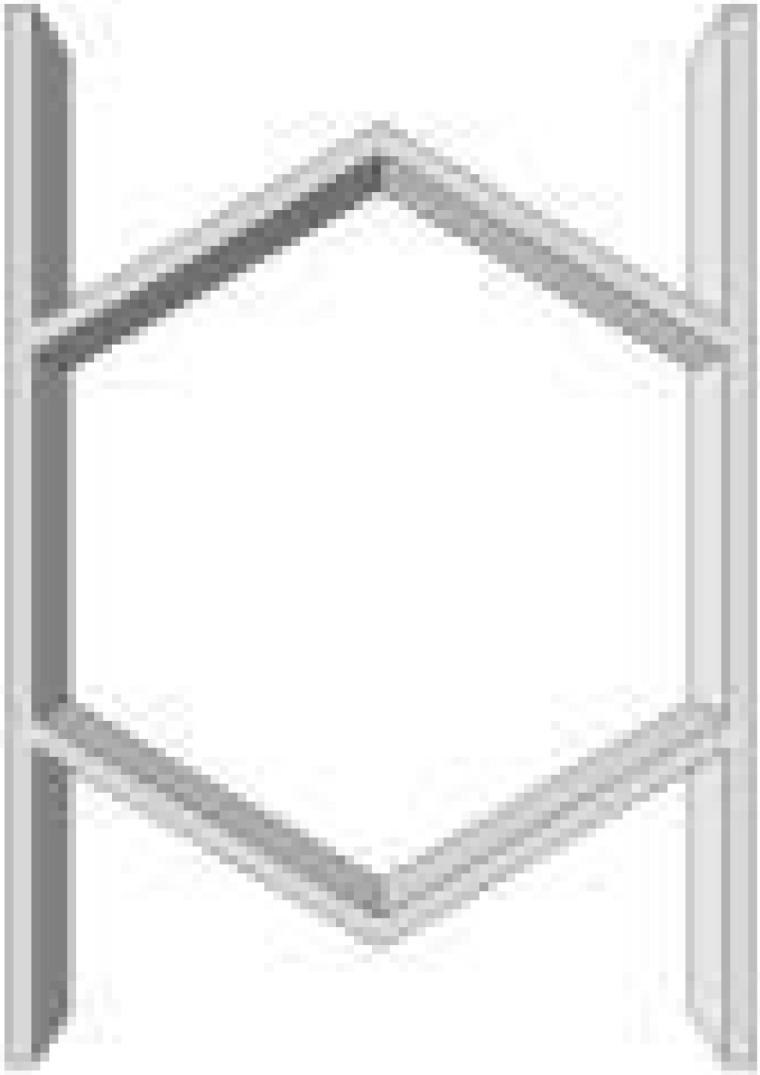	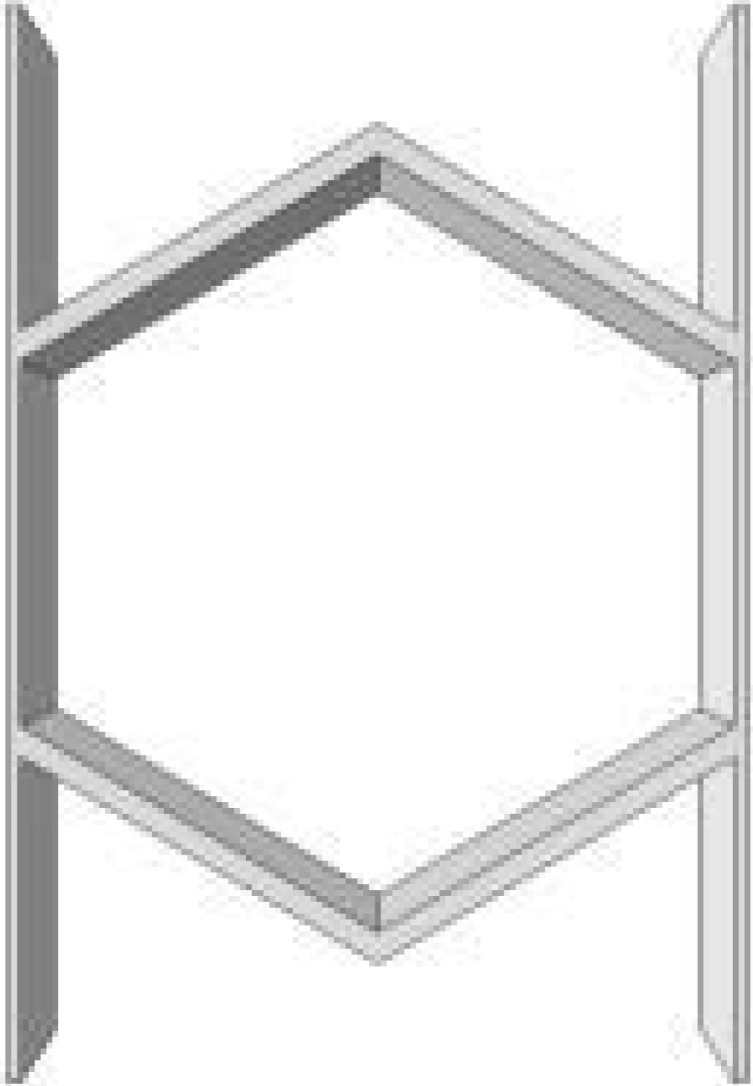

**Table 10:** Recommended parameter ranges for achieving performance targets based on FEM trends.

Performance Target	θ (°)	$l_{1}/t_{1}$	$l_{2}/l_{1}$	$l_{2}/t_{2}$
High elastic modulus $E_{1}$	70–80	<10	0.6–0.7	5–6.25
High elastic modulus $E_{2}$	60–70	<10	0.6–0.7	5–6.25
High shear modulus $G_{12}$	70–80	<10	0.6–0.7	5–6.25

Note: Ranges are approximate and derived from normalized trends shown in Figure 12.

## Data Availability

The original contributions presented in this study are included in the article. Further inquiries can be directed to the corresponding author.

## References

[1] Sun G., Chen D., Zhu G., Li Q. (2022). Lightweight hybrid materials and structures for energy absorption: A state-of-the-art review and outlook. Thin-Walled Struct..

[2] Gao Y., Li Z., Wei X., Du Y., Zhou Z., Xiong J. (2024). Advanced lightweight composite shells: Manufacturing, mechanical characterizations and applications. Thin-Walled Struct..

[3] Wu S., Zheng G., Sun G., Liu Q., Li G., Li Q. (2016). On design of multi-cell thin-wall structures for crashworthiness. Int. J. Impact Eng..

[4] Yeo S.J., Oh M.J., Yoo P.J. (2019). Structurally controlled cellular architectures for high-performance ultra-lightweight materials. Adv. Mater..

[5] Zhang Y., Liu G., Ye J., Lin Y. (2022). Crushing and parametric studies of polygonal substructures based hierarchical cellular honeycombs with non-uniform wall thickness. Compos. Struct..

[6] Correa D.M., Seepersad C.C., Haberman M.R. (2015). Mechanical design of negative stiffness honeycomb materials. Integr. Mater. Manuf. Innov..

[7] Lu H., Wang X., Chen T. (2021). In-plane dynamics crushing of a combined auxetic honeycomb with negative Poisson’s ratio and enhanced energy absorption. Thin-Walled Struct..

[8] Hu L.L., Zhou M.Z., Deng H. (2019). Dynamic indentation of auxetic and non-auxetic honeycombs under large deformation. Compos. Struct..

[9] Jiang H., Ren Y., Zhu G., Hu Y., Cheng F. (2020). Crashworthiness of novel concentric auxetic reentrant honeycomb with negative Poisson’s ratio biologically inspired by coconut palm. Thin-Walled Struct..

[10] Kalubadanage D., Remennikov A., Ngo T., Qi C. (2022). Experimental study on damage magnification effect of lightweight auxetic honeycomb protective panels under close-in blast loads. Thin-Walled Struct..

[11] Bohara R.P., Linforth S., Ghazlan A., Nguyen T., Remennikov A., Ngo T. (2022). Performance of an auxetic honeycomb-core sandwich panel under close-in and far-field detonations of high explosive. Compos. Struct..

[12] Huang J., Liu W., Tang A. (2018). Effects of fine-scale features on the elastic properties of zero Poisson’s ratio honeycombs. Mater. Sci. Eng. B.

[13] Liu W., Zhang Y., Guo Z., Li D., Zhao S., Xie W. (2023). Analyzing in-plane mechanics of a novel honeycomb structure with zero Poisson’s ratio. Thin-Walled Struct..

[14] Huang J., Zhang Q., Scarpa F., Liu Y., Leng J. (2017). Shape memory polymer-based hybrid honeycomb structures with zero Poisson’s ratio and variable stiffness. Compos. Struct..

[15] Simpson J., Kazancı Z. (2020). Crushing investigation of crash boxes filled with honeycomb and re-entrant (auxetic) lattices. Thin-Walled Struct..

[16] Wu P., Fu Y., Cai K. (2014). Regulation of the migration of endothelial cells by a gradient density of vascular endothelial growth factor. Colloids Surf. B.

[17] Song K., Li D., Zhang C., Liu T., Tang Y., Xie Y.M., Liao W. (2023). Bio-inspired hierarchical honeycomb metastructures with superior mechanical properties. Compos. Struct..

[18] Xu M., Zhao Z., Wang P., Duan S., Lei H., Fang D. (2022). Mechanical performance of bio-inspired hierarchical honeycomb metamaterials. Int. J. Solids Struct..

[19] Yuxuan L., Yifeng Z., Hien P.L., Yuxin T., Rong L. (2025). Gradient re-entrant honeycomb with quasi-ZPR and improved out-of-plane flexibility through tunable horizontal ligaments. Thin-Walled Struct..

[20] Liu W., Ma Y., Wang N., Luo Y., Tang A. (2022). A design of composite spar/shear web with ZPR honeycombs and graded structures for wind turbine blades. Mech. Adv. Mater. Struct..

[21] Grima J.N., Oliveri L., Attard D., Ellul B., Gatt R., Cicala G., Recca G. (2010). Hexagonal honeycombs with zero Poisson’s ratios and enhanced stiffness. Adv. Eng. Mater..

[22] Huang J., Zhang Q., Scarpa F., Liu Y., Leng J. (2016). Bending and benchmark of zero Poisson’s ratio cellular structures. Compos. Struct..

[23] Chen Y., Fu M. (2018). Mechanical properties of a novel zero Poisson’s ratio honeycomb. Adv. Eng. Mater..

[24] Broccolo S.D., Laurenzi S., Scarpa F. (2017). AUXHEX—A Kirigami inspired zero Poisson’s ratio cellular structure. Compos. Struct..

[25] Xu M.C., Xu Z.R., Zhang Z., Lei H., Bai Y., Fang D. (2019). Mechanical properties and energy absorption capability of AuxHex structure under in-plane compression: Theoretical and experimental studies. Int. J. Mech. Sci..

[26] Xu M.C., Liu D.B., Wang P.D., Zhang Z., Jia H., Lei H., Fang D. (2020). In-plane compression behavior of hybrid honeycomb metastructures: Theoretical and experimental studies. Aerosp. Sci. Technol..

[27] Liu K., Han L., Hu W.X., Ji L., Zhu S., Wan Z., Yang X., Wei Y., Dai Z., Zhao Z. (2020). 4D printed zero Poisson’s ratio metamaterial with switching function of mechanical and vibration isolation performance. Mater. Des..

[28] Wu H.X., Zhang X.C., Liu Y. (2020). In-plane crushing behavior of density graded cross-circular honeycombs with zero Poisson’s ratio. Thin-Walled Struct..

[29] Huang J., Gong X.B., Zhang Q.H., Scarpa F., Liu Y., Leng J. (2016). In-plane mechanics of a novel zero Poisson’s ratio honeycomb core. Compos. Part B Eng..

[30] Zadeh M.N., Dayyani I., Yasaee M. (2020). Fish Cells, a new zero Poisson’s ratio metamaterial-Part I: Design and experiment. J. Intell. Mater. Syst. Struct..

[31] Jha A., Dayyani I. (2021). Shape optimisation and buckling analysis of large strain zero Poisson’s ratio fish-cells metamaterial for morphing structures. Compos. Struct..

[32] Li Q., Yang D. (2024). A lightweight AuxHex zero Poisson’s ratio pressure-resistant sandwich cylindrical shell and its load-bearing and sound insulation behaviors: Design, simulation and experiment. Ocean Eng..

[33] Zhou Y., Ding Y., Pan Y., Wu J., Sun B., Gao Q. (2025). Multi-pattern crushing properties of coupling-designed honeycombs based on a non-contact interweaving combination strategy. Thin-Walled Struct..

[34] Zhang X., Zhu W., Tian R., Chen L., Guan H. (2024). Dual arrowhead-shaped re-entrant auxetic hybrid metamaterial with adjustable thermal expansion. Mater. Today Commun..

[35] Niknam H., Sarvestani H.Y., Jakubinek M.B., Ashrafi B., Akbarzadeh A.H. (2020). 3D printed accordion-like materials: A design route to achieve ultrastretchability. Addit. Manuf..

[36] Minfang C., Yifeng Z., Rong L., Shiwen W., Evrard I.A. (2024). Equivalent-oriented model for sandwich panels with ZPR accordion honeycomb. Int. J. Mech. Sci..

[37] Farrokhabadi A., Ashrafian M.M., Behravesh A.H., Hedayati S.K. (2022). Assessment of fiber-reinforcement and foam-filling in the directional energy absorption performance of a 3D printed accordion cellular structure. Compos. Struct..

[38] Rong L., Yifeng Z., Yilin Z., Haiwen C., Minfang C. (2024). Three-dimensional orthogonal accordion cellular structures with multi-directional zero Poisson’s ratio effects. Thin-Walled Struct..

[39] Wang Z., Li Z., Zhou W., Hui D. (2018). On the influence of structural defects for honeycomb structure. Compos. Part B Eng..

[40] Kahraman M.F., İriç S., Genel K. (2024). Comparative failure behavior of metal honeycomb structures under bending: A finite element-based study. Eng. Fail. Anal..

[41] Peng A., Deng J., Wu D., Zhang N., Guo Y., Cai D.A., Zhou G., Wang X. (2023). Damage behavior and failure mechanism of composite sandwich panel subjected to localized impact: A comprehensive study. Thin-Walled Struct..

[42] (2018). Standard Test Method for Tensile Properties of Plastics.

[43] Le Barbenchon L., Kopp J.-B. (2024). A review on the mechanical behaviour of microcellular and nanocellular polymeric foams: What is the effect of the cell size reduction?. J. Cell. Plast..

[44] Yifeng Z., Yu W. (2011). A variational asymptotic approach for hygrothermal analysis of composite laminates. Compos. Struct..

[45] Shi Z., Zhong Y., Yi Q., Peng X. (2021). High efficiency analysis model for composite honeycomb sandwich plate by using variational asymptotic method. Thin-Walled Struct..

